# A Domain-Specific Pretrained Model for Detecting Malignant and Premalignant Ocular Surface Tumors: A Multicenter Model Development and Evaluation Study

**DOI:** 10.34133/research.0711

**Published:** 2025-05-26

**Authors:** Zhongwen Li, Yangyang Wang, Wei Qiang, Xuefang Wu, Yanyan Zhang, Yiyuan Gu, Kuan Chen, Donghua Qi, Liheng Xiu, Yunduan Sun, Daoyuan Li, Yahui Xi, Shiqi Yin, Feng Wen, Mingmin Zhu, Yi Shao, Jiewei Jiang, Wei Chen, Guohai Wu

**Affiliations:** ^1^ Ningbo Key Laboratory of Medical Research on Blinding Eye Diseases, Ningbo Eye Institute, Ningbo Eye Hospital, Wenzhou Medical University, Ningbo 315040, China.; ^2^ National Clinical Research Center for Ocular Diseases, Eye Hospital, Wenzhou Medical University, Wenzhou 325027, China.; ^3^ Department of Pathology, The Affiliated People’s Hospital of Ningbo University, Ningbo 315100, China.; ^4^ Department of Ophthalmology, Cangnan Hospital, Wenzhou Medical University, Wenzhou 325000, China.; ^5^ Daqing Eye Hospital, Daqing 163711, China.; ^6^ Department of Ophthalmology, West China Second University Hospital, Sichuan University, Chengdu 610041, China.; ^7^ Department of Ophthalmology, The First Affiliated Hospital of Harbin Medical University, Harbin 150001, China.; ^8^ Department of Ophthalmology, The Affiliated Hospital of Guizhou Medical University, Guiyang 550004, China.; ^9^ Ankang Central Hospital, Xi’an Medical University, Xi’an 725000, China.; ^10^ School of Mathematics and Statistics, Xidian University, Xi’an 710071, China.; ^11^ Department of Ophthalmology, Shanghai General Hospital, Shanghai Jiao Tong University, National Clinical Research Center for Eye Disease, Shanghai 20080, China.; ^12^ School of Electronic Engineering, Xi’an University of Posts and Telecommunications, Xi’an 710121, China.

## Abstract

Malignant and premalignant ocular surface tumors (OSTs) can be sight-threatening or even life-threatening if not diagnosed and treated promptly. Artificial intelligence holds great promise for the early detection of these diseases. However, training traditional convolutional neural networks (CNNs) for this task presents challenges due to the lack of large, well-annotated datasets containing OST images labeled according to histopathological results. Here, we introduce the ocular surface pretrained model (OSPM), a domain-specific pretrained model designed to address the scarcity of labeled data. OSPM is constructed utilizing self-supervised learning on approximately 0.76 million unlabeled ocular surface images from 10 clinical centers across China and can be readily adapted to the OST classification task. We then develop and evaluate an OSPM-enhanced classification model (OECM) using 1,455 OST images labeled with histopathological diagnoses to differentiate between malignant, premalignant, and benign OSTs. OECM achieves excellent performance with AUROCs ranging from 0.891 to 0.993 on internal, external, and prospective test datasets, significantly outperforming the traditional CNN models. OECM demonstrated performance comparable to that of senior ophthalmologists and increased the diagnostic accuracy of junior ophthalmologists. Greater label efficiency was observed in OECM compared to CNN models. Our proposed model has high potential to enhance the early detection and treatment of malignant and premalignant OSTs, thereby reducing cancer-related mortality and optimizing functional outcomes.

## Introduction

Ocular surface tumors (OSTs) represent a large spectrum of conditions, including benign lesions such as corneoconjunctival dermoid, premalignant lesions such as conjunctival intraepithelial neoplasia (CIN), and life-threatening malignant lesions such as melanoma [1–3]. These tumors, frequently encountered in the clinical practice of cornea specialists, ocular oncologists, and comprehensive ophthalmologists [2], typically originate from epithelial, stromal, caruncular, and other tissues [3,4]. Notably, premalignant lesions may transform into malignant OSTs and malignant OSTs can lead to fatal metastases [1,2]. However, timely recognition and proper management of premalignant and malignant OSTs can improve the prognosis of patients, resulting in minimized cancer-related death and the most functionally satisfactory outcomes [2]. Therefore, accurately distinguishing the nature of OSTs at an early stage is paramount due to the substantial differences in their management.

Malignant, premalignant, and benign OSTs sometimes have overlapping clinical presentations [4,5]. Differentiating between them presents a clinical challenge and often requires experienced ophthalmologists [2,4]. The challenge of this task is exacerbated by a worldwide shortage of ophthalmologists, especially in underdeveloped regions [6]. Even in the United States, the total supply of full-time equivalent (FTE) ophthalmologists is projected to decline by 2,650 (12% drop) between 2020 and 2035, while demand is expected to increase by 5,150 FTE ophthalmologists (24% rise), indicating 30% mismatch between supply and demand that equates to a workforce inadequacy [7]. It is not practical to quickly solve this problem by increasing the training of ophthalmologists, as it takes at least 7 years to train an ophthalmic specialist [8]. For this reason, it is urgently needed to develop an approach that can discriminate among malignant, premalignant, and benign OSTs in an automated fashion.

Artificial intelligence (AI) holds great promise for widespread application in medical practice [9–11]. In ophthalmology, AI has demonstrated great potential as a primary tool for first-line medical care, particularly in scenarios where ophthalmologists are not readily accessible [12–14]. For instance, deep learning has shown robust performance in identifying diabetic retinopathy from fundus images [15,16], detecting keratitis from slit-lamp images [17,18], and recognizing wet age-related macular degeneration from optical coherence tomography images [19,20]. An automated deep learning model that can diagnose OSTs from ocular surface images may assist ophthalmologists in early discerning malignant and premalignant OSTs. However, the development of such an AI model has remained relatively underexplored.

In this study, we aim to establish an AI-assisted system to facilitate the rapid and robust diagnosis of malignant, premalignant, and benign OSTs. For the classification task, traditional deep learning models generally require substantial quantities of labeled data for effective training [13,21]. However, OSTs are rare ophthalmic conditions, with malignant and premalignant OSTs being even more uncommon. The scarcity of OST images annotated based on histopathological results cannot meet such an exhaustive requirement. To address the challenge of limited labeled data, we developed a domain-specific pretrained model, the ocular surface pretrained model (OSPM), using large quantities of unlabeled data to learn ocular surface feature representations that could enhance OST classification. Specifically, we trained OSPM with self-supervised learning (SSL) on approximately 0.76 million unlabeled ocular surface images. We then adapted OSPM for the diagnostic classification of OSTs (malignant, premalignant, and benign) by fine-tuning it with labeled OST images and evaluated the capability of the OSPM-enhanced classification model (OECM) on an external dataset of slit-lamp images. A dataset that included OST images captured by common digital cameras was utilized to verify the generalization ability of OECM. In addition, we conducted comparisons between OECM and models trained with other pretrained approaches, SSL methods, and convolutional neural network (CNN) architectures. Moreover, we compared the performance of OECM against ophthalmologists with various levels of expertise and explored the potential of OECM for assisting junior ophthalmologists in discriminating among malignant, premalignant, and benign OSTs.

## Results

### Data characteristics for developing OSPM

After excluding 4,132 poor-quality images, a total of 756,077 unlabeled ocular surface images collected from 10 independent clinical centers across China were used to construct OSPM. Detailed information on the data from each clinical center is provided in Table S1.

### Data characteristics for developing OECM

After excluding 666 OST images without histopathological results and 87 low-quality images, a total of 1,455 OST images from 840 patients [mean age, 43.6 years (range, 0.92 to 94), 45.3% women or girls] were used to establish and evaluate OECM. Specifically, OECM was trained and internally assessed using 669 images from Ningbo Eye Hospital (NEH). Then, it was externally evaluated on 298 images from the Eye Hospital of Wenzhou Medical University (EHWMU) and 351 images from Jiangdong Eye Hospital (JEH). Furthermore, OECM was assessed on 137 images prospectively acquired at NEH. The details of the datasets used for OECM development and evaluation are listed in Table S2.

The top 3 benign OSTs in our datasets are dermoid (179/914, 19.6%), nevus (167/914, 18.3%), and conjunctival cyst (149/914, 16.3%). Other benign OSTs, such as squamous epithelial papilloma, account for 45.8% (419/914). The top 3 malignant OSTs are squamous cell carcinoma (137/346, 39.6%), conjunctival lymphoma (73/346, 21.1%), and carcinoma in situ (CIS) (65/346, 18.8%). Other malignant OSTs, such as conjunctival melanoma, account for 20.5% (71/346). Our datasets contain 3 types of premalignant OSTs: primary acquired melanosis with atypia (149/195, 76.4%), CIN (37/195, 19.0%), and actinic keratosis (9/195, 4.6%).

### Performance of OSPM and other pretrained models

**Fig. 1.**
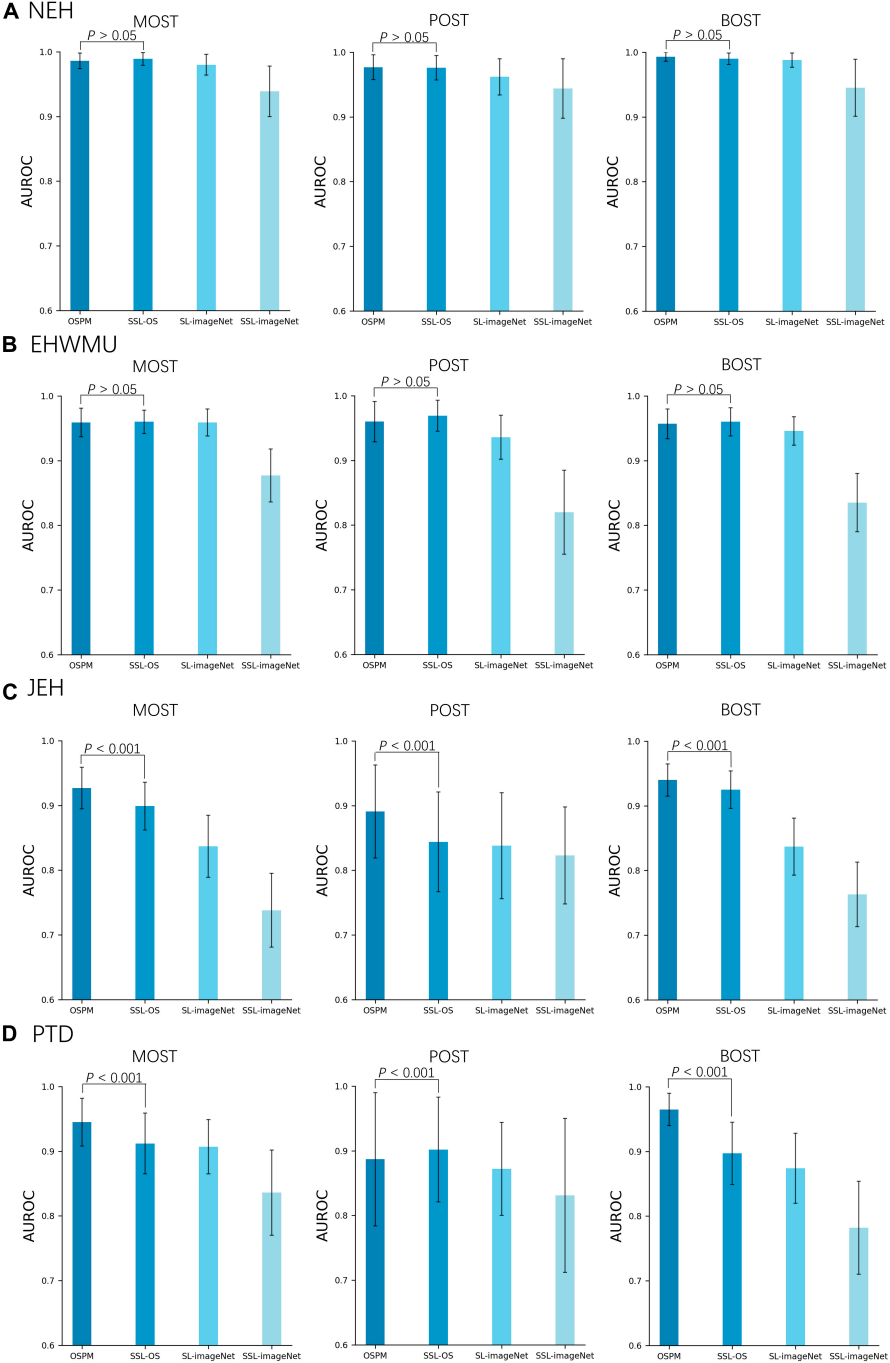
Performance of models using different pretraining approaches for the detection of malignant, premalignant, and benign ocular surface tumors (OSTs). (A) Internal test. Models are internally evaluated on OST images captured by slit-lamp imaging at Ningbo Eye Hospital (NEH). (B) External test. Models are externally evaluated on OST images captured by slit-lamp imaging at the Eye Hospital of Wenzhou Medical University (EHWMU). (C) External test. Models are externally evaluated on OST images captured by common digital cameras at Jiangdong Eye Hospital (JEH). (D) Prospective test. Models are prospectively evaluated on OST images captured by slit-lamp imaging at NEH. All models utilize different pretraining approaches but share the same architecture and fine-tuning processes for downstream tasks. The performance of OSPM is compared with the most competitive model to assess the presence of a statistically significant difference. AUROC, area under the receiver operating characteristic; PTD, prospective test dataset; MOST, malignant ocular surface tumor; POST, premalignant ocular surface tumor; BOST, benign ocular surface tumor; OSPM, ocular surface pretrained model; SSL, self-supervised learning; SL, supervised learning.

Among all pretrained models, the performance of OSPM and SSL-OS (ocular surface) ranked in the top 2 in all datasets (Fig. 1). Specifically, in the internal test set, OSPM achieved area under the receiver operating characteristic curves (AUROCs) of 0.986 [95% confidence interval (CI): 0.967 to 0.998], 0.977 (95% CI: 0.951 to 0.996), and 0.993 (95% CI: 0.980 to 1.000) for the identification of malignant, premalignant, and benign OSTs, respectively, similar to those of SSL-OS (all *P* > 0.05). In the EHWMU dataset (external evaluation), OSPM attained AUROCs of 0.959 (95% CI: 0.931 to 0.981), 0.960 (95% CI: 0.919 to 0.991), and 0.957 (95% CI: 0.928 to 0.980) for the identification of malignant, premalignant, and benign OSTs, respectively, comparable to those of SSL-OS (all *P* > 0.05). In the JEH dataset (external evaluation), OSPM reached AUROCs of 0.927 (95% CI: 0.892 to 0.959), 0.891 (95% CI: 0.797 to 0.963), and 0.940 (95% CI: 0.910 to 0.965) for the identification of malignant, premalignant, and benign OSTs, respectively, significantly better than those of SSL-OS (all *P* < 0.001). In the prospective test dataset, OSPM achieved AUROCs of 0.945 (95% CI: 0.898 to 0.982), 0.887 (95% CI: 0.749 to 0.990), and 0.965 (95% CI: 0.931 to 0.990) for the identification of malignant, premalignant, and benign OSTs, respectively, which were significantly better than those of SSL-OS, except for premalignant OSTs (all *P* < 0.001). The area under the precision–recall curve (AUPRC) results and corresponding confusion matrices of the models in these datasets are shown in Figs. S1 and S2, respectively. Further information on sensitivities, specificities, accuracies, and AUPRCs of the models is shown in Tables S3 to S6. Overall, OSPM outperformed other pretrained models in screening for malignant and premalignant OSTs. The unweighted Cohen’s κ values for the OSPM-based classification model, when compared to the reference standard of the internal test set, EHWMU dataset, JEH dataset, and prospective test dataset, were 0.834 (95% CI: 0.736 to 0.932), 0.771 (95% CI: 0.702 to 0.839), 0.781 (95% CI: 0.717 to 0.845), and 0.840 (95% CI: 0.758 to 0.923), respectively.

### Performance of different SSL approaches

**Fig. 2.**
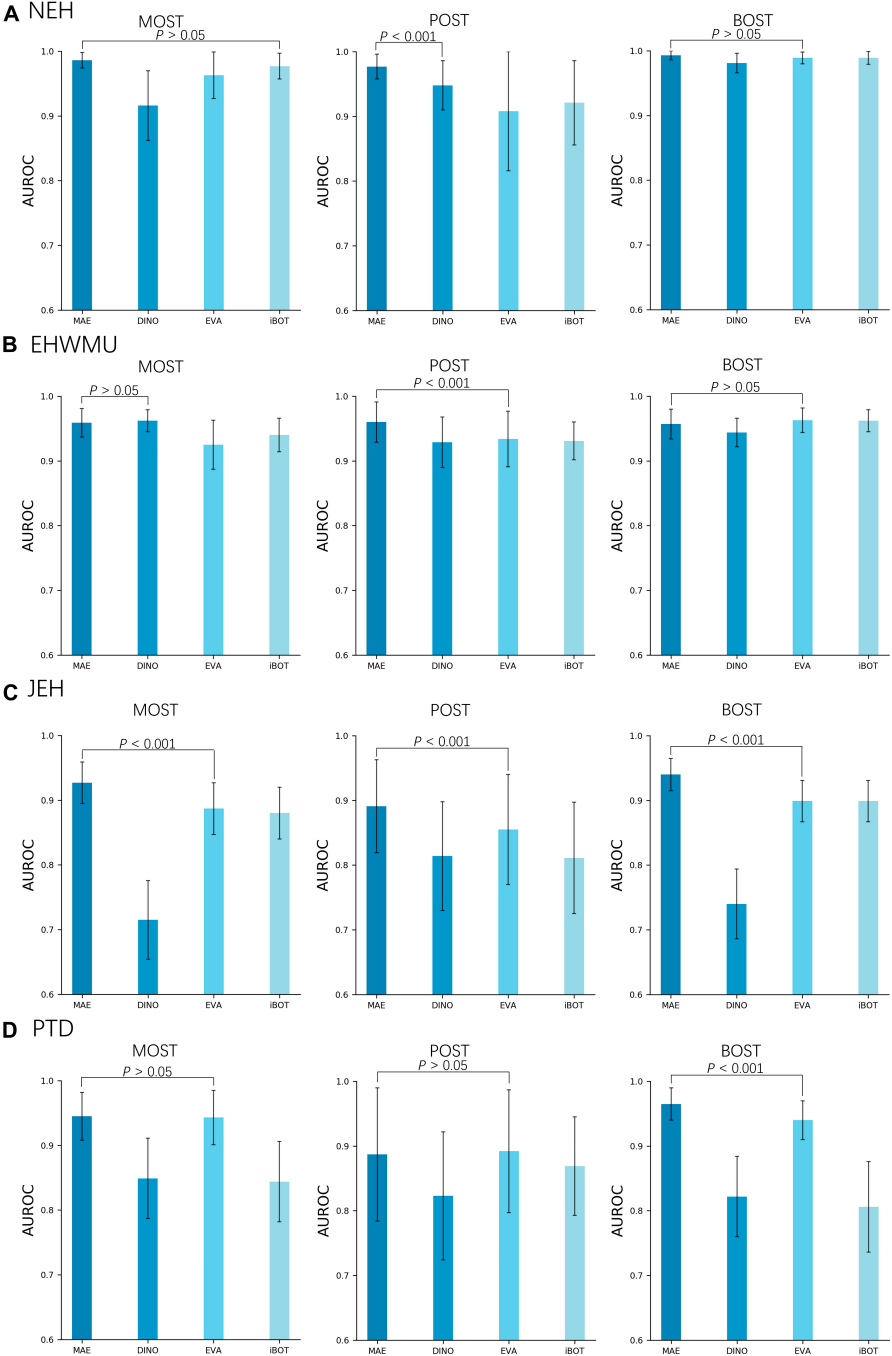
Performance of models using different SSL approaches for the detection of malignant, premalignant, and benign OSTs. (A) Internal test. Models are internally evaluated on OST images captured by slit-lamp imaging at NEH. (B) External test. Models are externally evaluated on OST images captured by slit-lamp imaging at the EHWMU. (C) External test. Models are externally evaluated on OST images captured by common digital cameras at JEH. (D) Prospective test. Models are prospectively evaluated on OST images captured by slit-lamp imaging at NEH. Models pretrained with different SSL approaches, including masked autoencoder (MAE), DINO, EVA, and iBOT, undergo the same fine-tuning processes for downstream tasks. The performance of the OSPM, pretrained with MAE, is compared with the most competitive model to assess the presence of a statistically significant difference.

In the OSPM framework, we investigated the performance of various SSL approaches, including masked autoencoder, DINO, EVA, and iBOT. As illustrated in Fig. 2 and Figs. S3 and S4, the masked autoencoder, the main SSL strategy in the OSPM framework, significantly outperformed other approaches in the majority of OST detection tasks. A detailed listing of all quantitative results can be found in Tables S7 to S10.

### OECM versus CNN models

**Fig. 3.**
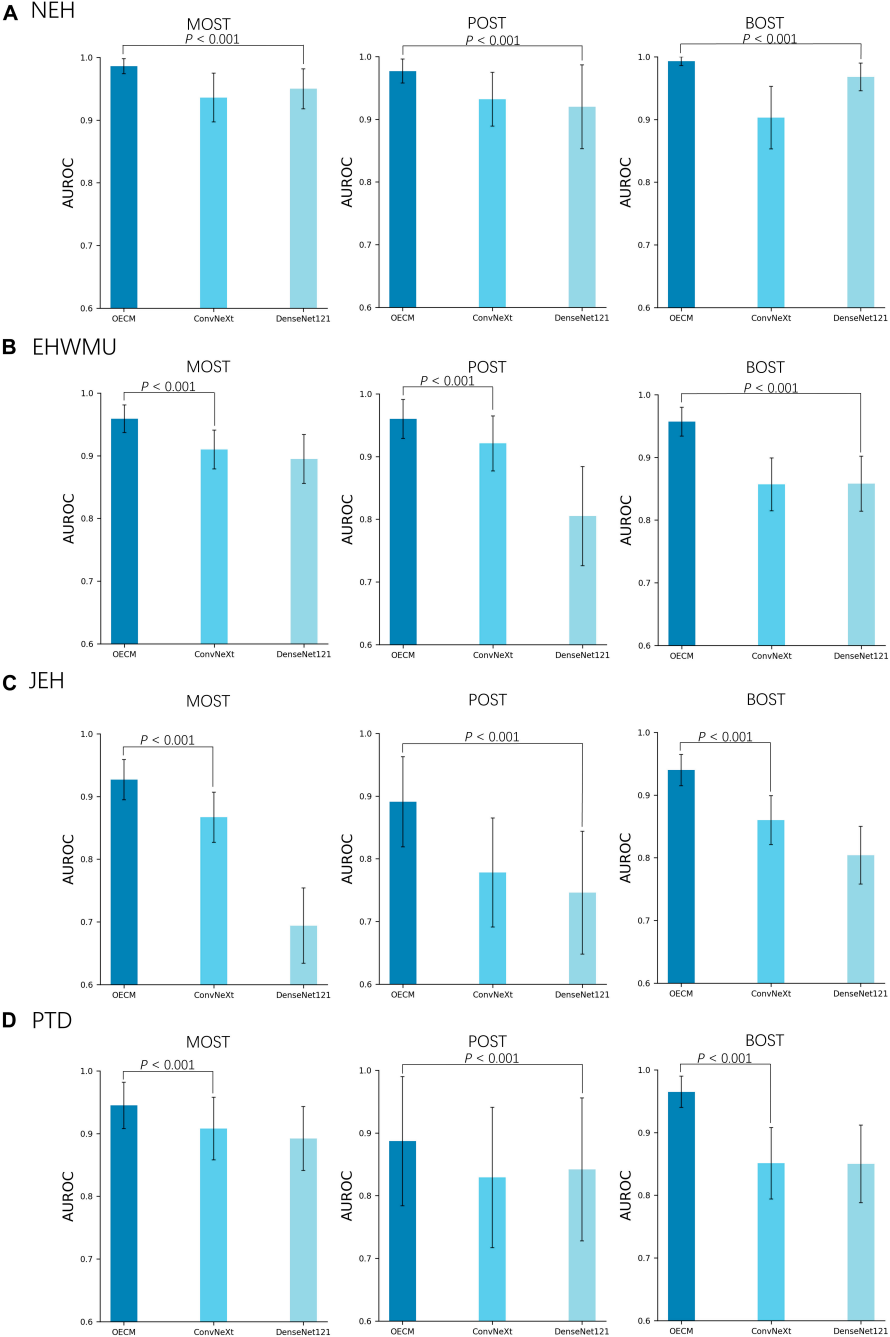
Performance comparison of the OSPM-enhanced classification model (OECM) and CNN models in detecting malignant, premalignant, and benign OSTs. (A) Internal test. Models are internally evaluated on OST images captured by slit-lamp imaging at NEH. (B) External test. Models are externally evaluated on OST images captured by slit-lamp imaging at the EHWMU. (C) External test. Models are externally evaluated on OST images captured by common digital cameras at JEH. (D) Prospective test. Models are prospectively evaluated on OST images captured by slit-lamp imaging at NEH. The performance of OECM is compared with the most competitive model to assess the presence of a statistically significant difference.

Compared to CNN models, OECM demonstrated superior performance across all datasets (Fig. 3). For example, on discriminating malignant OSTs from premalignant and benign OSTs, OECM achieved AUROC of 0.986 (95% CI: 0.967 to 0.998), 0.959 (95% CI: 0.931 to 0.981), 0.927 (95% CI: 0.892 to 0.959), and 0.945 (95% CI: 0.898 to 0.982), respectively, in the internal test, EHWMU, JEH, and prospective test datasets, significantly outperforming ConvNeXt and DenseNet121 (all *P* < 0.001). OECM also achieved consistently high performance in the detection of premalignant and benign OSTs. In terms of AUPRC, OECM significantly outperformed the CNN models (Fig. S5). The confusion matrices of the models in these datasets are shown in Fig. S6. A detailed listing of all quantitative results is presented in Tables S11 to S14. In the JEH external test set (OST images captured by common digital cameras instead of slit-lamp imaging), both OECM and traditional CNN models showed varying degrees of performance degradation, with AUROCs ranging from 0.694 to 0.940. It is worth noting that among these models, OECM has the smallest performance drop.

As depicted in Fig. S7, all models exhibited similar convergence speeds, with training loss stabilizing after 22 epochs. Notably, OECM achieved a lower final training loss than those of the 2 traditional CNN models.

### Label efficiency of OECM and CNN models

**Fig. 4.**
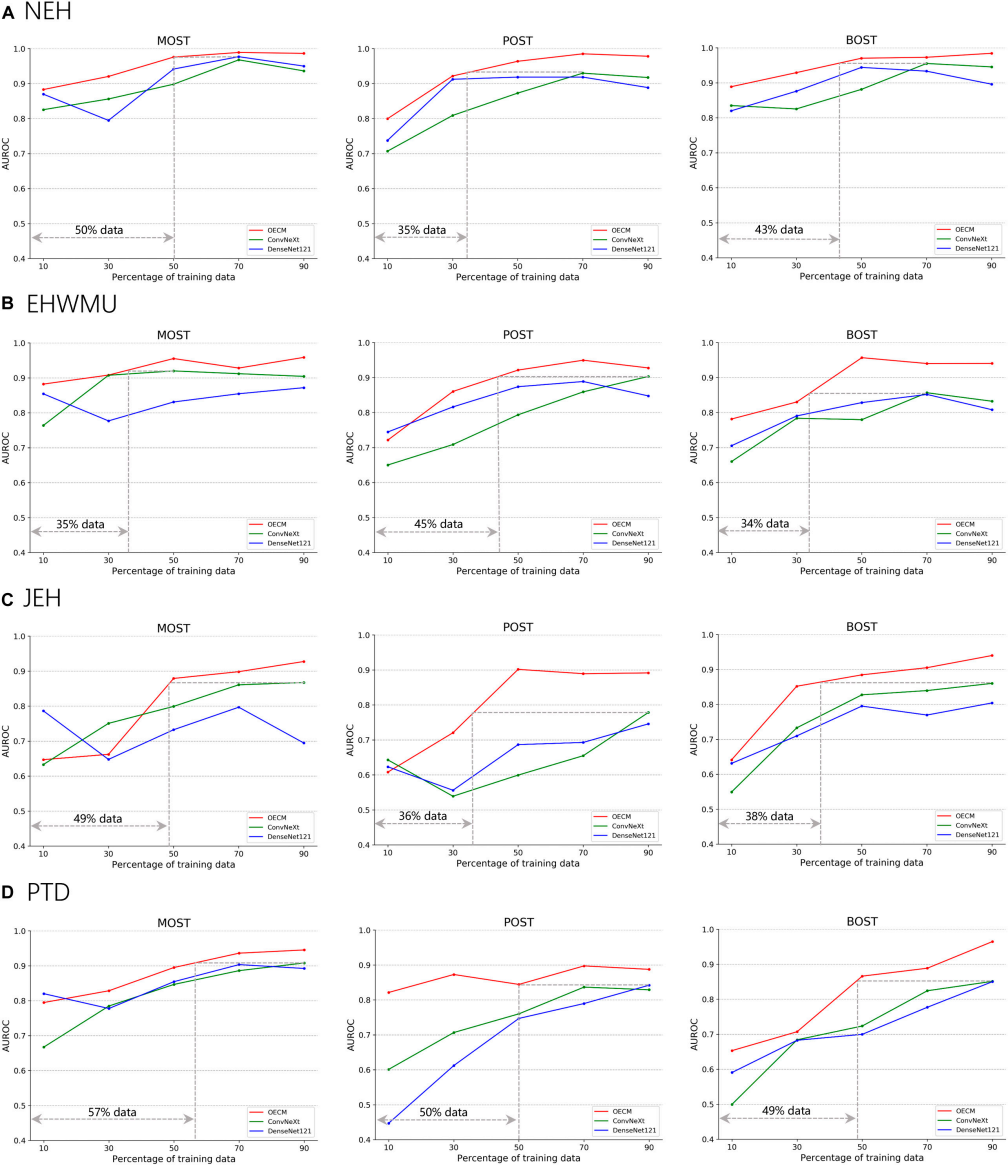
Label efficiency of the OECM and CNN models in detecting malignant, premalignant, and benign OSTs. Label efficiency refers to the quantity of labeled training data required to achieve optimal performance for a specified downstream task. The dashed gray line indicates the disparity in training data between OECM and the most competitive CNN model used for comparison. (A) Label efficiency of the models on the NEH dataset. (B) Label efficiency of the models on the EHWMU dataset. (C) Label efficiency of the models on the JEH dataset. (D) Label efficiency of the models on the prospective test dataset.

As shown in Fig. 4, OECM exhibited higher label efficiency than that of CNN models in identifying malignant, premalignant, and benign OSTs. In detecting malignant OSTs, OECM outperformed CNN models with only 35% to 57% of the labeled training data, indicating its potential to address the challenges of limited data availability. OECM also demonstrated superior label efficiency in detecting both premalignant and benign OSTs. In addition, the training efficiency (defined as the epochs necessary for the model to reach training convergence) of OECM was higher than that of the CNN models, indicating that OECM required fewer epochs to adapt to the downstream task (Fig. S8). To be specific, OECM can reduce the number of training epochs by 10 to 13 to reach convergence in differentiating between malignant, premalignant, and benign OSTs.

### Classification errors of OECM

The comparative analysis revealed a discordance between the OECM outputs and the reference standard in 101 of 889 images (11.4%) across all 3 validation datasets (internal, external, and prospective). In the class of malignant OSTs (241 images), 14 images (5.8%) were misclassified by OECM as premalignant tumors, and 14 images (5.8%) were misclassified as benign tumors. For the malignant OSTs incorrectly classified as premalignant, 50.0% (7/14) images are CIS located at the limbus. These cases share similar features with CIN, which may have contributed to the misclassification. For the malignant OSTs misclassified as benign, 57.1% (8/14) of the images show tumors located at the conjunctival fornix with inadequate exposure. In the class of premalignant OSTs (112 images), 12 images (10.7%) were misclassified by OECM as malignant tumors, and 6 images (5.4%) were misclassified as benign tumors. For the premalignant OSTs misclassified as malignant, 58.3% (7/12) of the images show CIN with features similar to CIS. For the premalignant OSTs misclassified as benign, the most common reason is the presence of a small tumor near the limbus, observed in 50.0% (3/6) of the images. In the class of benign OSTs (536 images), 26 images (4.9%) were misclassified by OECM as malignant tumors and 29 images (5.4%) were misclassified as premalignant tumors. For the benign OSTs incorrectly classified as malignant, more than half of the images (65.4%, 17/26) show conjunctival hyperemia around the tumor. For the benign OSTs misclassified as premalignant, 51.7% (15/29) of the images show relatively large tumors near the inner or outer canthus. Representative examples of misclassified images are presented in Fig. S9.

As depicted in Fig. S10, a strong inverse relationship was observed between the predicted probability scores of the OECM and the corresponding classification error rates. The figure demonstrates that both the error rates for individual categories and the total classification error rate rise as the predicted probabilities diminish. When the predicted probabilities exceed 0.87, the misclassification rates of malignant and premalignant OSTs are around 10%, while the misclassification rate of benign OSTs is approximately 5%. The analysis revealed that classification error rates exceeded 20% when predicted probabilities fell below 0.6. As a 3-class classifier, OECM maintains a minimum predicted probability threshold of >0.33 for all outputs.

### Interpretability of OSPM and OECM

**Fig. 5.**
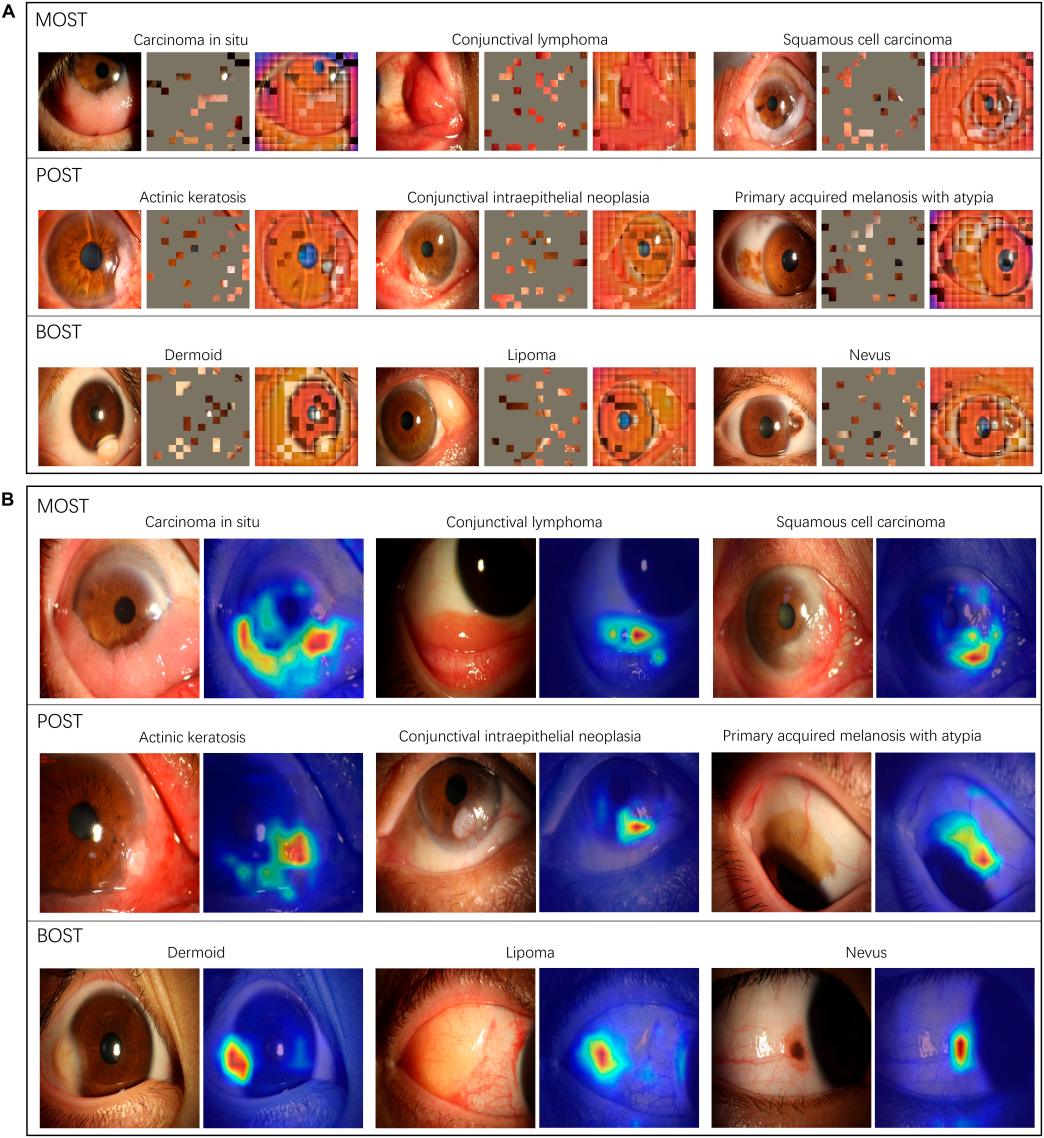
Visual interpretation of the OSPM and the OECM. (A) Reconstructed ocular surface images from highly masked photographs in the pretext task. Despite the limited visibility of the patches, OSPM successfully infers ocular surface anatomical structures (e.g., the cornea and conjunctiva), as well as tumors, which serve as markers for the subsequent classification task. (B) Heatmaps highlighting the regions that contribute most to the decisions made by OECM. The tumor areas in the images, irrespective of their location, shape, or size, are detected and utilized for classification.

To understand the mechanisms behind OSPM’s superior performance and label efficiency in the OST classification task, qualitative analyses were conducted on the pretext task utilized for self-supervised pretraining, as well as on the task-specific decisions made by OSPM. The pretext task in OSPM enables models to capture the distinct context of the ocular surface, integrating knowledge of both anatomical structures and lesions. As depicted in Fig. 5A, OSPM successfully reconstructed key anatomical structures (such as the cornea and conjunctiva) and tumors, even with 75% of the ocular surface image masked. This illustrates that OSPM has acquired the ability to discern and infer representations of disease-related regions through SSL, thereby improving both classification performance and label efficiency in OST diagnosis. Furthermore, to generate interpretable visual explanations of the OECM’s classification decisions for malignant, premalignant, and benign OSTs, we employed the RELPROP technique to create discriminative heatmaps. These heatmaps consistently highlighted tumor regions in the images, regardless of their location, shape, or size. Representative heatmap visualizations for malignant, premalignant, and benign OSTs are shown in Fig. 5B.

### OECM versus ophthalmologists

We conducted a comparative evaluation of the OECM against practicing ophthalmologists using a balanced contest dataset consisting of 150 images randomly selected from external test datasets (50 malignant, 50 premalignant, and 50 benign OSTs). Four board-certified ophthalmologists participated in the study, stratified into 2 experience-based groups: junior (6 to 15 years of clinical experience) and senior (16 to 25 years of clinical experience) practitioners.

**Fig. 6.**
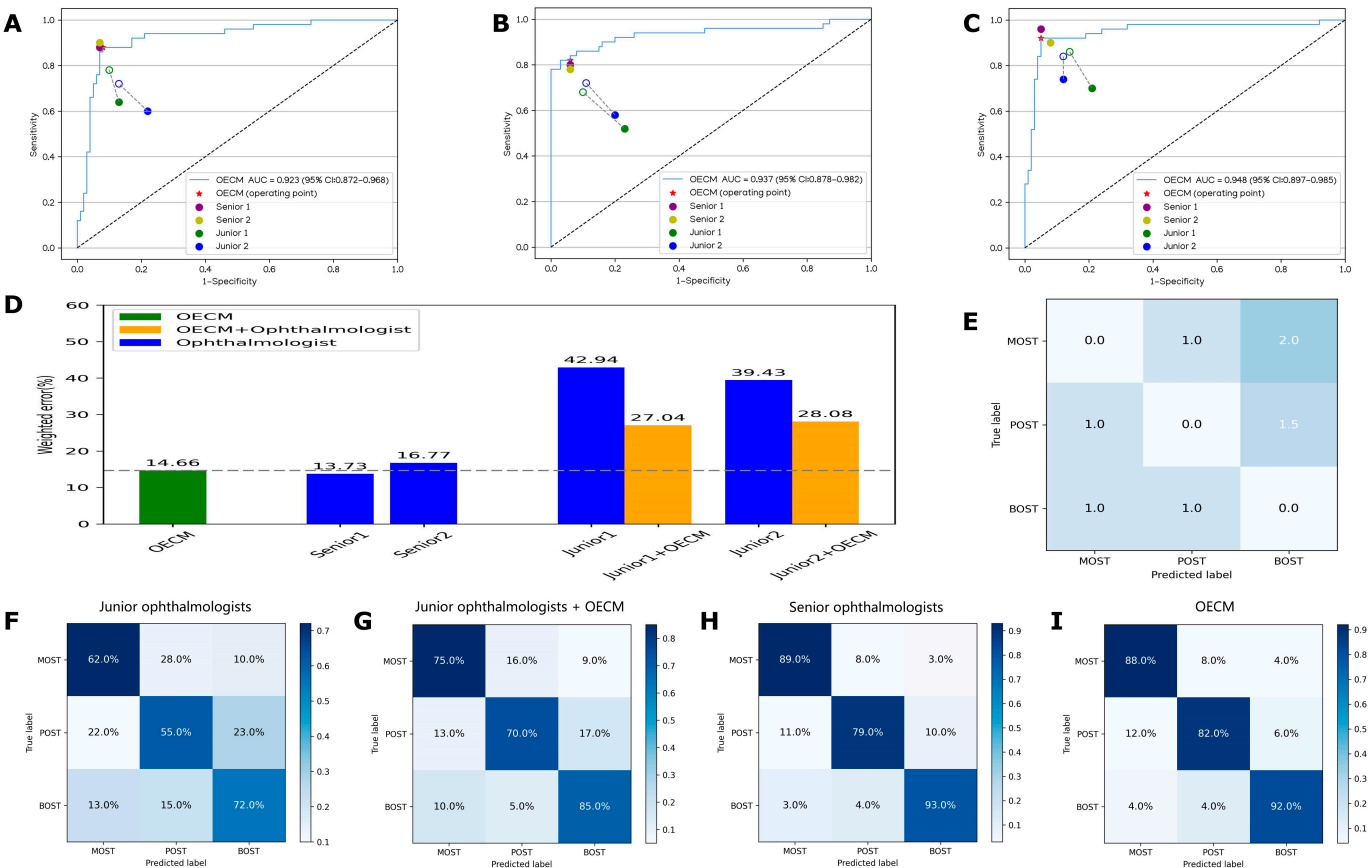
Diagnostic performance comparisons between the OECM and ophthalmologists. (A to C) Performance of OECM and 4 ophthalmologists (2 junior and 2 senior). Receiver operating characteristic (ROC) curves for identifying the different types of OSTs. Filled dots represent the performance of junior and senior ophthalmologists, while hollow dots depict the performance of junior ophthalmologists assisted by OECM. Dashed lines connect the performance metrics of each junior ophthalmologist in pairs. (A) ROC curve for the diagnosis of MOST versus other categories. (B) ROC curve for the diagnosis of POST versus other categories. (C) ROC curve for the diagnosis of BOST versus other categories. (D) Weighted errors calculated using penalty scores. (E) Penalty scoring matrix. (F to I) Confusion matrices of 3-category classification. (F) Confusion matrix representing the average performance of 2 junior ophthalmologists. (G) Confusion matrix representing the average performance of 2 junior ophthalmologists with OECM assistance. (H) Confusion matrix representing the average performance of 2 senior ophthalmologists. (I) Confusion matrix representing the performance of OECM.

Overall, OECM demonstrated significantly better diagnostic performance than junior ophthalmologists while achieving non-inferior results compared to senior specialists in OST classification. The comprehensive performance comparison between OECM and the ophthalmologists is detailed in Table S15. Additionally, we leveraged predicted errors, based on penalty scores (Fig. 6D and E), to create a metric to assess and compare performance between OECM and the ophthalmologists. OECM yielded a weighted error of 14.7% compared to a range of weighted errors from the ophthalmologists, which varied from 13.7% to 42.9%, with a mean of 28.2% (Fig. 6D).

To evaluate OECM’s potential as a diagnostic decision-support tool, we provided junior ophthalmologists with the model’s probability outputs (Fig. S11) for each test image and assessed subsequent performance improvements. To avoid a potential memorization bias, the follow-up OECM-assisted diagnostic test conducted by the ophthalmologists was scheduled 4 weeks after the initial test. With the aid of OECM, the performance of the junior ophthalmologists showed a marked enhancement over their previous results, as illustrated in Fig. 6. Specifically, the assistance of OECM significantly increased the accuracies of junior ophthalmologist 1 in identifying premalignant and benign OSTs (*P* < 0.05) and improved the accuracies of junior ophthalmologist 2 in detecting malignant and premalignant OSTs (*P* < 0.05), as presented in Table S15.

## Discussion

This study was designed to develop a robust AI-based diagnostic system for detecting malignant and premalignant OSTs from ocular surface images captured using multiple commercial digital cameras. The key finding of this study was that our OECM, built upon OSPM—a novel SSL-based domain-specific pretrained model established using 0.76 million unlabeled ocular surface images from 10 independent clinical centers across China—demonstrated excellent performance in the OST classification task. In external and prospective test datasets, the sensitivities for identifying malignant and premalignant OSTs ranged from 80.0% to 90.9%, while the specificities ranged from 91.9% to 95.9%, demonstrating the robustness of OECM. In addition, the unweighted Cohen’s κ coefficients indicated substantial agreement (all κ > 0.77) between OECM classifications and the reference standard, confirming the system’s diagnostic reliability. Given its reliable performance, OECM has the potential to assist ophthalmologists in the early detection of malignant and premalignant OSTs, thereby facilitating timely medical intervention.

The OSPM-based model (OECM) and the SSL-OS-based model ranked in the top 2 across all datasets for discriminating among malignant, premalignant, and benign OSTs (Fig. 1). Both OSPM (which applied SSL to natural and ocular surface images) and SSL-OS (which used SSL exclusively on ocular surface images) were fine-tuned using labeled OST images captured via slit-lamp imaging. In the NEH internal test dataset and the EHWMU external test dataset, both comprising slit-lamp images, these 2 models demonstrated comparable performance in the OST classification task. However, in the JEH external dataset, which consists of OST images acquired using standard digital cameras, the OSPM-based classification model demonstrated significantly superior performance compared to the SSL-OS-based model. This result illustrates that incorporating natural images along with ocular surface images for SSL can improve the model’s adaptability to heterogeneous capture devices.

In the JEH external test set, both the OECM and traditional CNN models exhibited varying levels of performance degradation, with AUROCs ranging from 0.694 to 0.940 (Table S13). Notably, the OECM model experienced the smallest decline in performance among the models tested. This indicates that OECM demonstrates superior generalization and broader recognition capabilities compared to traditional AI approaches that rely on CNNs trained with supervised learning methods.

Compared to the CNN models, OECM demonstrated higher label efficiency in identifying malignant, premalignant, and benign OSTs (Fig. 4). Specifically, OECM achieved better performance than the CNN models while using only 35% to 50% of the labeled training data, highlighting its potential to overcome the challenges associated with limited data availability. In addition, the training efficiency of OECM was also superior to that of the CNN models. This illustrates that OECM can decrease the number of training epochs by 10 to 13 to achieve convergence in the OST classification task (Fig. S8), particularly when utilizing optimization techniques such as early stopping.

OECM sustained competitive performance in the OST classification task, even when different contrastive SSL methods were integrated into the framework (Fig. 2 and Fig. S3). The masked autoencoder outperformed other approaches such as DINO, EVA, and iBOT. The comparative analysis demonstrated that the ocular surface-specific representations learned through masked autoencoding—which effectively encoded anatomical structures (cornea, conjunctiva) and pathological features (tumors) (Fig. 5A)—provided critical discriminative power for differentiating between malignant, premalignant, and benign OSTs.

To assess the clinical performance of OECM, we compared it with ophthalmologists of varying experience levels. Our findings demonstrated that the OECM achieved diagnostic parity with senior ophthalmologists while significantly enhancing the accuracy of junior ophthalmologists (Fig. 6). This dual capability positions the OECM as a valuable decision-support tool, potentially enhancing diagnostic efficiency in overburdened healthcare systems requiring rapid diagnostic triage and in underserved regions with limited access to specialist ophthalmologists.

Although OECM demonstrated robust performance, misclassifications were still observed. Quantitative analysis revealed a negative correlation between OECM’s predicted probabilities and misclassification rates, indicating that cases with lower confidence scores had substantially higher likelihood of diagnostic error (Fig. S10). Therefore, cases with low predicted probability scores (<0.6) should be flagged for physician review to ensure diagnostic accuracy. An optimal AI diagnostic system should minimize both false positives and false negatives while preserving clinical utility. Further research is warranted to elucidate the mechanisms underlying errors and to refine model performance.

Recently, Yoo et al. [22] trained a model using low-shot deep learning to identify patients with conjunctival melanoma, nevus, pterygium, and normal conjunctival based on 398 raw ocular surface images and 400 synthetic images created through generative adversarial networks. The accuracy of the model reached 87.5% in this 4-class classification task. Since their model was specifically optimized for conjunctival melanoma detection, its generalizability to other malignant OSTs may be limited. Taki et al. [23] developed an intelligent system named CorneAI, which was based on the YOLO V5 architecture and used 357 anterior segment images to detect corneal diseases, including corneal tumors. Although the positive predictive value of CorneAI in detecting corneal tumors was 0.77, it cannot differentiate the nature of the tumor. Compared to previous studies, our research presents several important features. First, to our knowledge, we developed the first SSL-based domain-specific model, OSPM, using over half a million unlabeled ocular surface images and leveraged it to enhance the OECM in distinguishing malignant, premalignant, and benign OSTs. The ground-truth labels for all images in the OECM development dataset were obtained from definitive histopathological diagnoses. OECM presented robust performance in images collected from multiple clinical centers using various imaging devices. In addition, OECM performed effectively on images captured with ordinary digital cameras. This denotes that OECM can be used with such cameras, providing a cost-effective and convenient way for high-risk groups to proactively detect malignant and premalignant OSTs through self-photography. Third, we confirmed that OECM exhibited higher label efficiency and training efficiency compared to the CNN models. This indicates that OECM may substantially reduce the manual annotation workload for specialists, perform well in tasks with limited data, and offer excellent fine-tuning efficiency. Fourth, we validated that OECM improved junior ophthalmologists’ ability to differentiate OSTs, which may lead to more rapid and effective treatment planning for premalignant and malignant cases.

The current study has several limitations. First, OECM was developed using data from Chinese cohorts. Its effectiveness in other racial populations needs further verification. Additional training with data from diverse demographic cohorts could enhance the model’s performance across a wider range of populations. Moreover, the human–AI comparative analysis was limited to single-image evaluations, which offer substantially less diagnostic information than comprehensive clinical assessments that incorporate patient history and physical examination. However, this experimental design intentionally matched the informational constraints of OECM, thereby enabling a rigorous, controlled comparison between human and AI performance under equivalent conditions. Future research could explore the development of multimodal AI systems incorporating clinical history, comprehensive examination findings, and diverse imaging modalities to improve diagnostic precision.

In conclusion, our study developed OECM that showed excellent performance and broad generalizability in distinguishing between malignant, premalignant, and benign OSTs. As a preliminary screening tool, OECM could potentially be deployed with both specialized slit-lamp cameras and ordinary digital cameras, enabling the early detection of malignant and premalignant OSTs. This approach may help patients receive timely referrals and effective medical interventions, reducing cancer-related mortality and maximizing functional outcomes.

## Methods

### Study approval

Our study protocol received ethical approval from the NEH Ethics Committee (identifier, 2022-49K-SB34) and was undertaken in accordance with the Declaration of Helsinki guidelines. All photographic data (file sizes: 0.2 to 9 MB per image) were stripped of any patient-related information before researchers accessed them. The NEH Ethics Committee granted a waiver for informed consent for the use of retrospectively collected images, whereas informed consent was secured for images collected prospectively. The study was conducted in compliance with the CONSORT-AI extension guidelines, the recently developed standards for AI-related clinical research [24].

### Datasets

For the development of OSPM, we used 756,077 unlabeled ocular surface images that were retrospectively collected from 10 clinical centers across China using a variety of imaging devices. For the establishment of OECM, we utilized 1,455 OST images that were labeled according to definitive histopathological confirmation. The images were acquired across multiple clinical environments (outpatient, inpatient, and surgical settings), exhibiting natural variations in illumination and background conditions that enhanced the dataset’s diversity and clinical representativeness. Images were considered low quality if they met any of the following criteria: (a) more than one-fifth of the ocular surface was covered by the eyelids; (b) the image was not focused on the ocular surface; or (c) more than one-fifth of the ocular surface appeared blurred due to misalignment with the imaging plane. All low-quality images were initially excluded from the study by our previously established AI-based image quality monitoring system [25]. Detailed information on datasets utilized in this study is described in Tables S1 and S2.

### OSPM development

Data preprocessing was conducted prior to the development of OSPM. First, we resized all unlabeled slit-lamp images to 224 × 224 pixels using cubic interpolation. Next, to enhance the diversity of the training dataset, mitigate bias, and improve the model’s generalization capability, we applied identical data augmentation techniques used for masked autoencoder training [26] to all images, including (a) random resized cropping, (b) horizontal flipping, and (c) pixel value normalization.

We developed OSPM using a unique configuration of the masked autoencoder, consisting of both an encoder and a decoder. The architecture of OSPM is illustrated in Fig. S12. The encoder architecture utilized a large vision Transformer (ViT-large) comprising 24 transformer blocks with 1,024-dimensional embeddings, while the decoder implemented a smaller variant (ViT-small) with 8 transformer blocks and 512-dimensional embeddings. Unmasked 16 × 16 pixels were transformed by the encoder into 1,024-dimensional latent space vectors. The 24 Transformer blocks, each containing multi-head self-attention mechanisms and multilayer perceptrons (MLPs), progressively transformed these feature vectors into higher-level representations. In the decoding phase, masked placeholder patches were combined with the encoded high-level features, which were then transformed through a linear projection layer to reconstruct the image patches. The training objective focused on reconstructing ocular surface images from heavily masked inputs, challenging the model to recover morphological details from limited visual information. The detailed parameter settings for developing OSPM are provided in Table S16. We preserved the network weights from the concluding epoch as the reference checkpoint for transfer learning to downstream applications (i.e., construction of the OSPM-enhanced model for discriminating among malignant, premalignant, and benign OSTs). Six NVIDIA Tesla A40 GPUs (44 GB each) were used to develop OSPM.

### Construction of OECM

The reference standard for each OST image used in OECM development is determined by an unequivocal histopathological diagnosis. All cases were classified by the study steering committee according to the latest World Health Organization (WHO) diagnostic criteria (5th edition, 2023) [27], establishing 3 diagnostic categories: (a) malignant OSTs, (b) premalignant OSTs, and (c) benign OSTs. Malignant OSTs included squamous cell carcinoma, conjunctival melanoma, conjunctival lymphoma, etc. Premalignant OSTs encompassed CIN, primary acquired melanosis with atypia, conjunctival actinic keratosis, etc. Benign OSTs comprised conjunctival squamous papilloma, nevus, conjunctival epithelial cyst, etc.

**Fig. 7.**
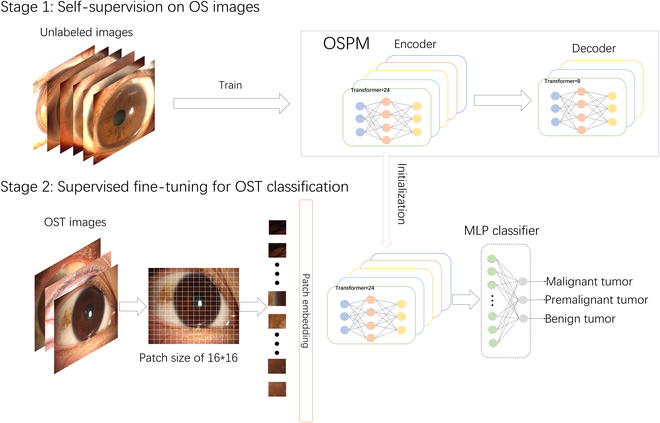
Overview of the OECM. In stage 1, OSPM is constructed using masked autoencoders trained on 0.76 million unlabeled ocular surface (OS) images. The detailed architecture of OSPM is illustrated in Fig. S12. In stage 2, OSPM is adapted to the OST classification task through supervised learning. The OECM consists of a pretrained transformer encoder and a multilayer perceptron (MLP) head, with the encoder initialized from OSPM.

In constructing OECM for identifying malignant, premalignant, and benign OSTs, we only utilized the encoder of the OSPM, which could create high-level features from ocular surface images. These features were fed into an MLP, which then outputted the probability for each disease category. The final diagnostic classification was assigned based on the highest predicted probability among all candidate categories. The details of OECM are described in Fig. 7.

For OECM development, we randomly allocated the OST images from the NEH dataset following a stratified 70:15:15 ratio for training, validation, and internal testing respectively. The data splitting was performed at the patient level to prevent any potential data leakage. All model training was conducted on a computational cluster equipped with 4 NVIDIA GeForce RTX 3090 GPUs (CUDA 11.3) supported by an Intel Xeon Gold 6248R CPU (3.00 GHz). All computational operations were performed within an Ubuntu 20.04 LTS environment equipped with 754 GB of system memory. Each input image was preprocessed and standardized to a resolution of 224 × 224 pixels. The same data augmentation procedures used in OSPM development were applied to enhance training dataset diversity. The model was trained for 50 epochs with a batch size of 32. Model optimization was performed using stochastic gradient descent with an initial learning rate of 0.01 and a weight decay coefficient of 1 × 10^−4^. The model was evaluated on the validation set after every epoch, with weights corresponding to the peak AUROC retained as the optimal checkpoint.

The performance of OECM was assessed on OST images captured by digital slit-lamp imaging at EHWMU and on OST images captured by common digital cameras at JEH. These 2 external datasets provided a stringent test of the OECM’s generalization ability, as they included images from different patients, imaging conditions, and devices not encountered in the development dataset. We performed a prospective pilot study at NEH between June 2024 and October 2024 to further evaluate the performance of OECM in a clinical setting. The development and evaluation workflow of OECM is shown in Fig. S13.

### Performance of different pretrained models

We compared the performance of OSPM with 3 other pretrained models: SL-ImageNet, SSL-ImageNet, and SSL-OS. Despite varying pretraining approaches, all models had the same structure and downstream task fine-tuning procedures. SL-ImageNet utilized supervised pretraining on ImageNet-1k (1.3 million labeled natural images), whereas SSL-ImageNet employed SSL on the same dataset without labels. In contrast, SSL-OS applied self-supervised pretraining directly to ocular surface images. OSPM initialized with SSL-ImageNet weights before fine-tuning on ocular surface images, mirroring the 2-stage self-supervised pretraining approach (natural images followed by domain-specific images). The pretraining process of each model is illustrated in Fig. S14.

### Comparison between different SSL approaches

To investigate the performance of utilizing different SSL approaches, we modified the OSPM architecture by integrating DINO, EVA, and iBOT as alternative SSL strategies, replacing the baseline masked autoencoder. This step generated a range of variations of the pretrained model for comparative analysis. The network architectures and hyperparameters were adopted from the respective original studies to ensure the optimal performance of each contrastive learning method. The models were initialized with ImageNet-1k pretrained weights, followed by contrastive learning-based pretraining on 760,000 unlabeled ocular surface images. Subsequently, we applied the same fine-tuning protocol used for the masked autoencoder to adapt these pretrained models for the OST classification task.

### Performance comparison of OECM with CNN models

In recent years, CNNs have shown remarkable performance in disease diagnosis from medical images [13]. A comparative performance analysis was conducted between OECM and other CNN architectures using both internal and external test sets. We used ConvNeXt and DenseNet121, pretrained using supervised learning on ImageNet-1k (approximately 1.3 million labeled natural images), as representative models.

### Label efficiency comparison between OECM and CNN models

Label efficiency quantifies the minimum amount of annotated training data required to achieve optimal performance on a target task, directly reflecting the annotation burden on clinical experts [28]. To demonstrate the superior label efficiency of OECM in OST classification, we compared it with that of CNN models (ConvNeXt and DenseNet121) on both internal and external test sets.

### Interpretations for OECM

To ensure that human experts trust OECM, a transparent decision-making process is essential in clinical practice. RELPROP [29], a technique that employs layer-wise relevance propagation to calculate and integrate relevance scores for each attention head across the attention graph, was used to provide promising explanations for model decisions. As a result, a heatmap of attention was generated to highlight the regions that OECM referenced when performing OST classification.

### Misclassification by OECM

We conducted a post hoc evaluation of OECM’s performance, enumerating misclassified images by OST type and analyzing the nature of classification errors. To elucidate these discrepancies, we systematically analyzed image characteristics underlying misclassifications and documented potential causative factors. Additionally, we investigated the association between prediction errors and the system’s output probability values.

### Performance comparison between OECM and ophthalmologists

To compare OECM’s performance against ophthalmologists, we assembled a comparative dataset of 150 images (50 malignant, 50 premalignant, and 50 benign OST cases) randomly sampled from external test datasets. These images were shuffled and anonymized before evaluation by the ophthalmologists. Four practicing ophthalmologists (independent of image annotation) were recruited and stratified into 2 cohorts: junior (6 to 15 years’ experience) and senior (16 to 25 years’ experience) groups. Each ophthalmologist independently classified the images into malignant, premalignant, or benign OST categories based solely on visual assessment, without access to any ancillary clinical data. To preclude competition bias, the ophthalmologists were not informed about their comparison with the AI system. Moreover, to eliminate the influence of prevalence disparities among various OST types, they were notified that the dataset might not reflect the true distribution of different OST types in real-world clinical settings. We used a weighted error based on a penalty score to evaluate the clinical performance of OECM and the ophthalmologists. Misdiagnosis of malignant and premalignant OST as benign OST was assigned scores of 2 and 1.5, respectively, as these errors could lead to the worst outcomes. In contrast, misdiagnosing benign OST as malignant or premalignant OST was assigned a score of 1. In addition, a score of 1 was consistently assigned to all additional classification errors.

Subsequently, we designed a complementary study to evaluate OECM’s potential to augment diagnostic performance among junior ophthalmologists. This involved the reevaluation of the same images 4 weeks after their initial inspection, with OECM providing the predicted probabilities for different types of OST for each image. The ophthalmologists were then instructed to reaffirm their diagnoses, thereby assessing the influence of OECM on their diagnostic proficiency by comparing their findings with the reference standard.

### Statistical analyses

To assess the models’ performance, we used sensitivity, specificity, accuracy, AUROC, and AUPRC. These metrics were calculated using the one-versus-rest strategy, where the performance of each category was compared against all other categories. Specifically, a multiclass categorization issue was disaggregated into a sequence of binary classification problems. These assessments evaluated the model’s capability to differentiate each class from the remaining classes. For all performance metrics, we calculated 95% CIs to quantify the estimated uncertainty embedded within the metrics. All plots were created with the following tools: Matplotlib v3.5.1, Seaborn v0.11.2, and R v4.2.1. The DeLong test [30], a statistical method designed for quantifying the differences between 2 AUROCs derived from an identical pool of individuals, was employed to compare the AUROC values of the models. We employed the McNemar test [31] to evaluate differences in diagnostic performance metrics (sensitivity, specificity, and accuracy) both between models and between OECM and clinical experts. The degree of agreement between OECM outputs and the reference standard was quantified using unweighted Cohen’s κ values, with interpretation based on established benchmarks: ≤0 (no agreement), 0.01 to 0.20 (slight), 0.21 to 0.40 (fair), 0.41 to 0.60 (moderate), 0.61 to 0.80 (substantial), and 0.81 to 1.00 (almost perfect agreement). All statistical analyses were performed using Python 3.7.8, with statistical significance defined as a 2-tailed *P* value < 0.05.

## Data Availability

The main data supporting the findings of this study are available within the article and its Supplementary Materials. For privacy reasons, the raw datasets from each hospital cannot be publicly disclosed. However, researchers can request access to a subset of the data for academic purposes, contingent on the approval of the corresponding author and the institutional review boards of the respective hospitals. The code used to train, fine-tune, and evaluate the models in this study is publicly accessible at https://github.com/NBeye-research/OSPM.
