# Supplementary material for: A Domain-Specific Pretrained Model for Detecting Malignant and Premalignant Ocular Surface Tumors: A Multicenter Model Development and Evaluation Study
Source: Research (Wash D C). 2025 May 26;8:0711. doi: 10.34133/research.0711 (PMC12104561; doi:10.34133/research.0711)
Supplement: Supplementary 1 — Figs. S1 to S14 Tables S1 to S16 [file research.0711.f1.docx]

**Supplementary Materials**

**A Domain-Specific Pretrained Model for Detecting Malignant and Premalignant Ocular Surface Tumors: A Multicenter Model Development and Evaluation Study**

**Figs. S1 to S14**

**Tables S1 to S16**


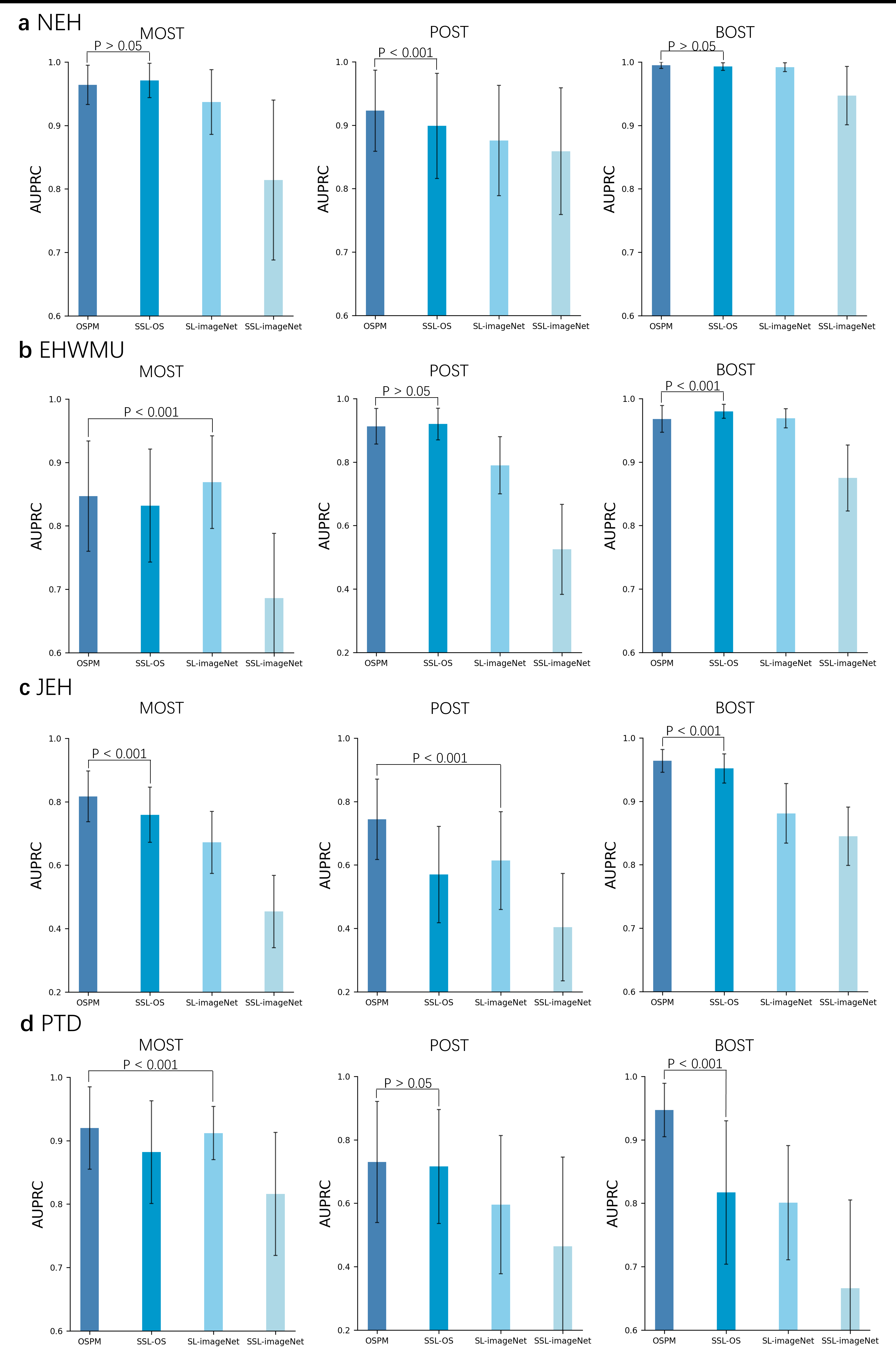


**Fig. S1. Performance (AUPRC) of models using different pretraining approaches for the detection of malignant, premalignant, and benign OSTs.** **a** Internal test. Models are internally evaluated on OST images captured by slit-lamp imaging at NEH. **b** External test. Models are externally evaluated on OST images captured by slit-lamp imaging at EHWMU. **c** External test. Models are externally evaluated on OST images captured by common digital cameras at JEH. **d** Prospective test. Models are prospectively evaluated on OST images captured by slit-lamp imaging at NEH. All models utilize different pretraining approaches but share the same architecture and fine-tuning processes for downstream tasks. The performance of OSPM is compared with the most competitive model to assess the presence of a statistically significant difference. AUPRC, area under the precision-recall curve. NEH, Ningbo Eye Hospital. EHWMU, Eye Hospital of Wenzhou Medical University. JEH, Jiangdong Eye Hospital. PTD, prospective test dataset. MOST, malignant ocular surface tumor. POST, premalignant ocular surface tumor. BOST, benign ocular surface tumor. OSPM, ocular surface pretrained model. SSL, Self-supervised learning. SL, supervised learning.


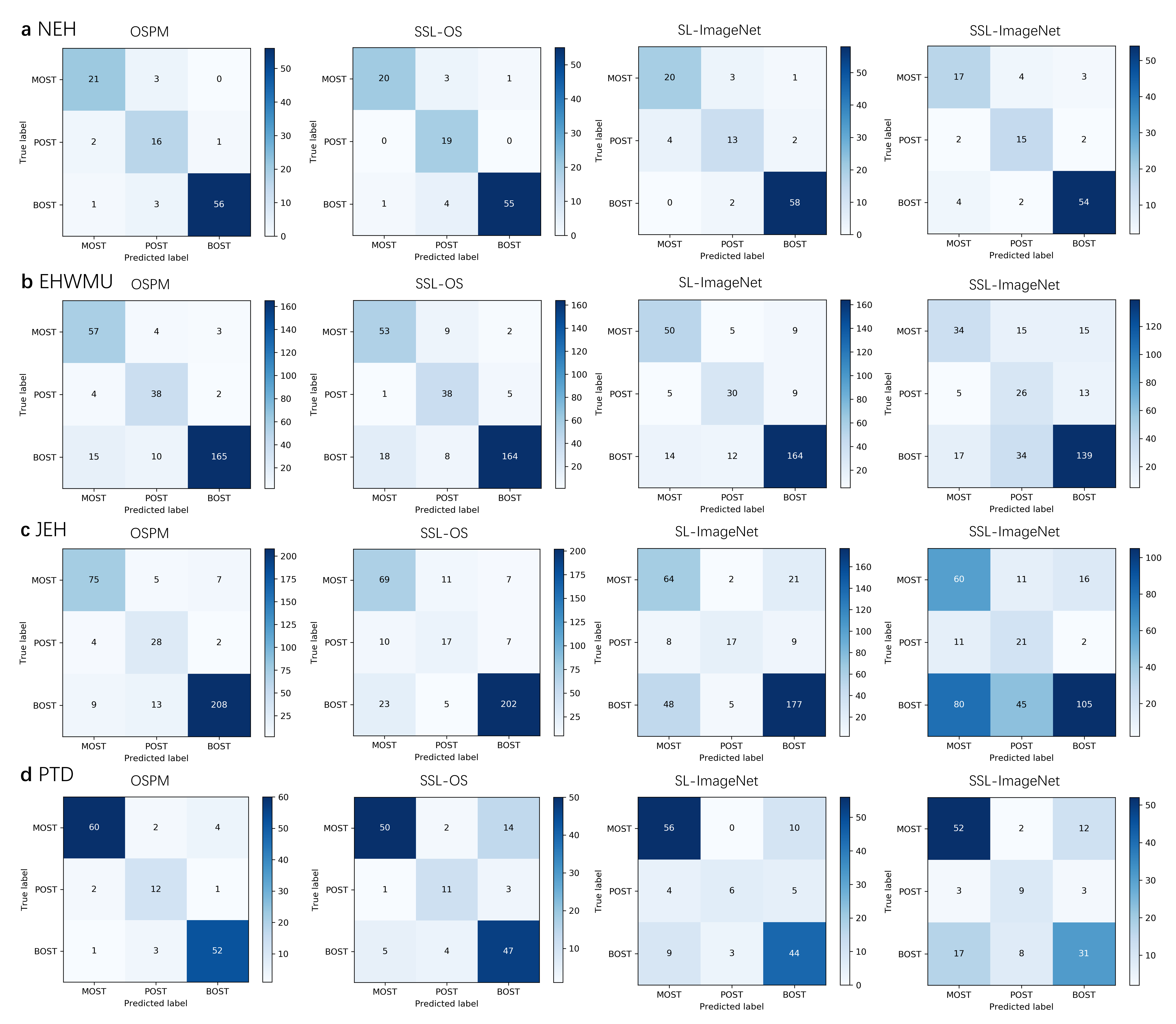


**Fig. S2. Confusion matrices of models using different pretraining approaches for the classification of malignant, premalignant, and benign OSTs.** **a** Internal test. Models are internally evaluated on OST images captured by slit-lamp imaging at NEH. **b** External test. Models are externally evaluated on OST images captured by slit-lamp imaging at EHWMU. **c** External test. Models are externally evaluated on OST images captured by common digital cameras at JEH. **d** Prospective test. Models are prospectively evaluated on OST images captured by slit-lamp imaging at NEH. NEH, Ningbo Eye Hospital. EHWMU, Eye Hospital of Wenzhou Medical University. JEH, Jiangdong Eye Hospital. PTD, prospective test dataset. MOST, malignant ocular surface tumor. POST, premalignant ocular surface tumor. BOST, benign ocular surface tumor. OSPM, ocular surface pretrained model. SSL, Self-supervised learning. SL, supervised learning.

**
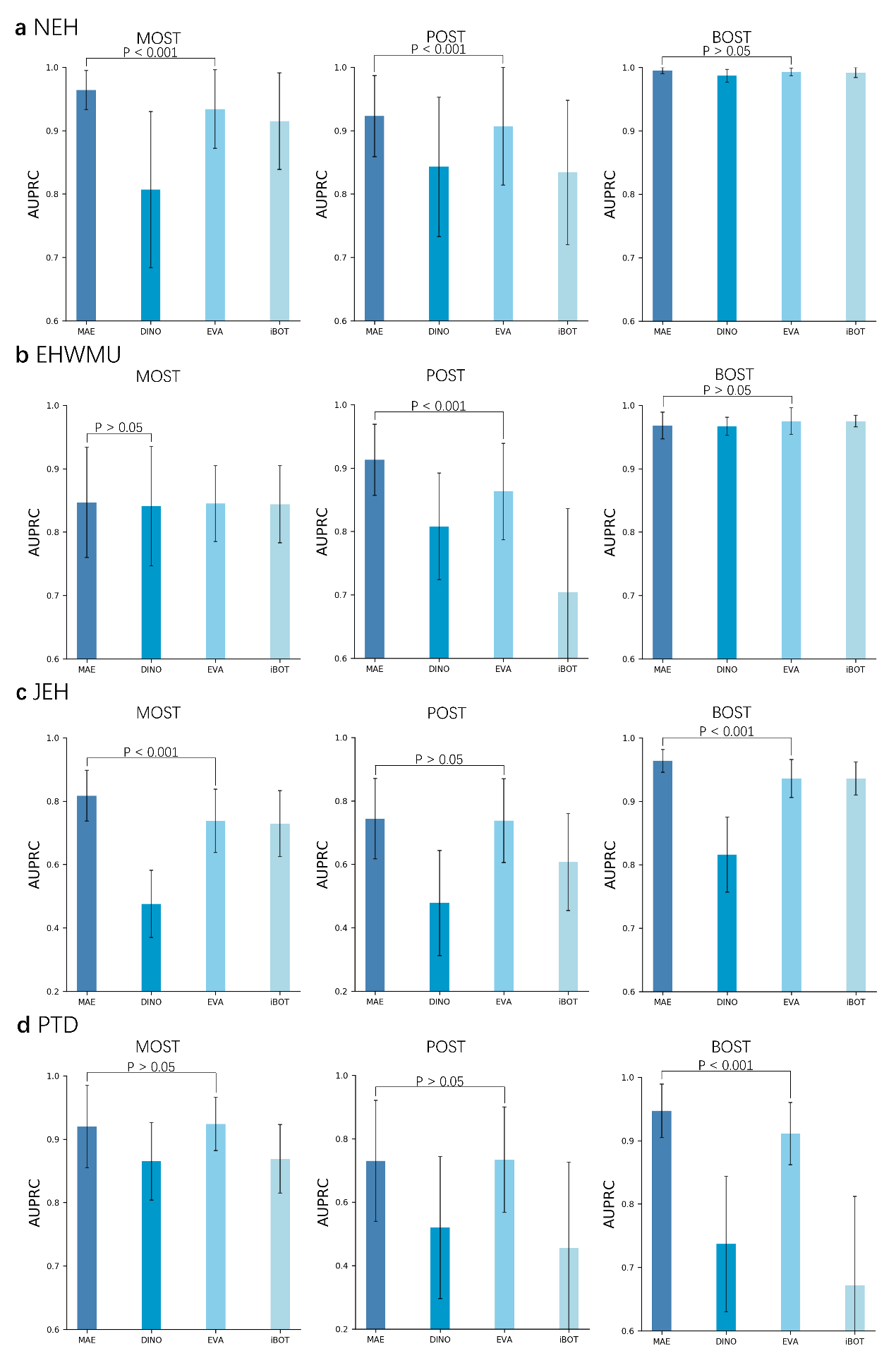
**

**Fig. S3. Performance (AUPRC) of models using different SSL approaches for the detection of malignant, premalignant, and benign OSTs. a** Internal test. Models are internally evaluated on OST images captured by slit-lamp imaging at NEH. **b** External test. Models are externally evaluated on OST images captured by slit-lamp imaging at EHWMU. **c** External test. Models are externally evaluated on OST images captured by common digital cameras at JEH. **d** Prospective test. Models are prospectively evaluated on OST images captured by slit-lamp imaging at NEH. Models pretrained with different SSL approaches, including MAE, DINO, EVA, and iBOT, undergo the same fine-tuning processes for downstream tasks. The performance of OSPM (pretrained with MAE) is compared with the most competitive model to assess the presence of a statistically significant difference. AUPRC, area under the precision-recall curve. NEH, Ningbo Eye Hospital. EHWMU, Eye Hospital of Wenzhou Medical University. JEH, Jiangdong Eye Hospital. PTD, prospective test dataset. MOST, malignant ocular surface tumor. POST, premalignant ocular surface tumor. BOST, benign ocular surface tumor. OSPM, ocular surface pretrained model. MAE, Masked Autoencoders. SSL, Self-supervised learning.


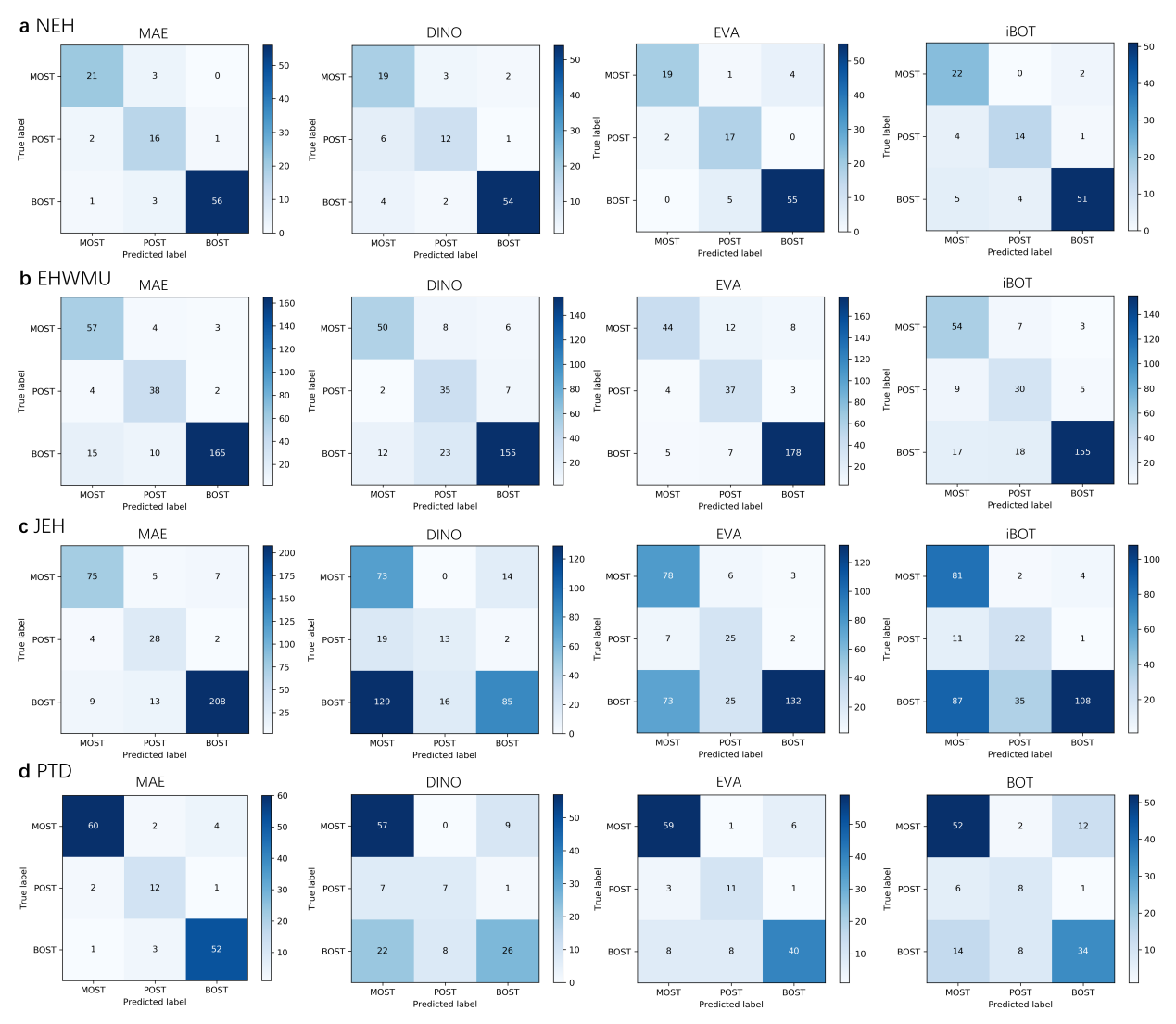
 **Fig. S4. Confusion matrices of models using different SSL approaches for the classification of malignant, premalignant, and benign OSTs.** **a** Internal test. Models are internally evaluated on OST images captured by slit-lamp imaging at NEH. **b** External test. Models are externally evaluated on OST images captured by slit-lamp imaging at EHWMU. **c** External test. Models are externally evaluated on OST images captured by common digital cameras at JEH. **d** Prospective test. Models are prospectively evaluated on OST images captured by slit-lamp imaging at NEH. NEH, Ningbo Eye Hospital. EHWMU, Eye Hospital of Wenzhou Medical University. JEH, Jiangdong Eye Hospital. PTD, prospective test dataset. MOST, malignant ocular surface tumor. POST, premalignant ocular surface tumor. BOST, benign ocular surface tumor. MAE, Masked Autoencoders. SSL, Self-supervised learning.

**
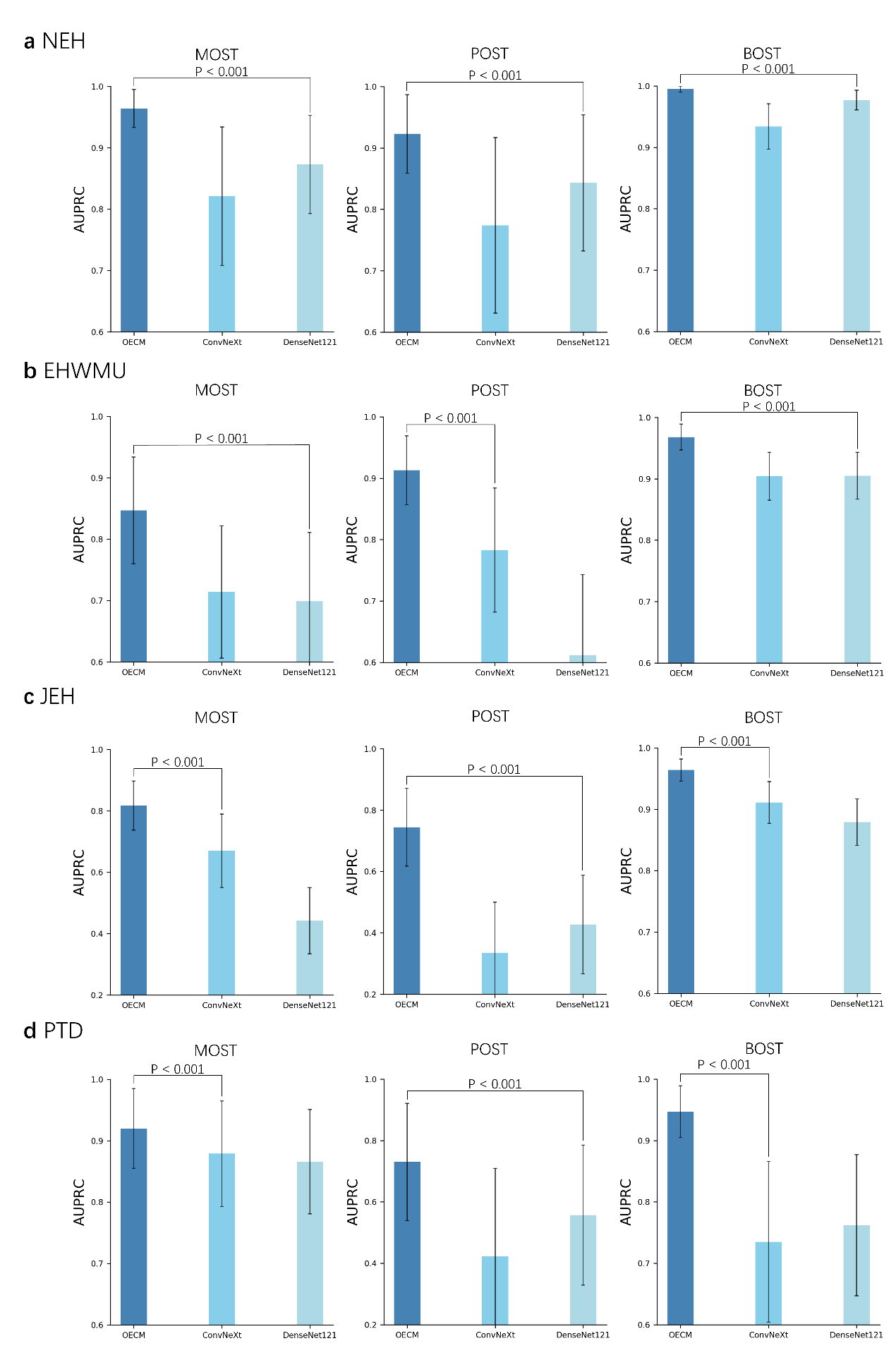
**

**Fig. S5.** **Performance (AUPRC) comparison of OECM and CNN models in detecting malignant, premalignant, and benign OSTs. a** Internal test. Models are internally evaluated on OST images captured by slit-lamp imaging at NEH. **b** External test. Models are externally evaluated on OST images captured by slit-lamp imaging at EHWMU. **c** External test. Models are externally evaluated on OST images captured by common digital cameras at JEH. **d** Prospective test. Models are prospectively evaluated on OST images captured by slit-lamp imaging at NEH. The performance of OECM is compared with the most competitive model to assess the presence of a statistically significant difference. AUPRC, area under the precision-recall curve. NEH, Ningbo Eye Hospital. EHWMU, Eye Hospital of Wenzhou Medical University. JEH, Jiangdong Eye Hospital. PTD, prospective test dataset. MOST, malignant ocular surface tumor. POST, premalignant ocular surface tumor. BOST, benign ocular surface tumor. OECM, OSPM-enhanced classification model.


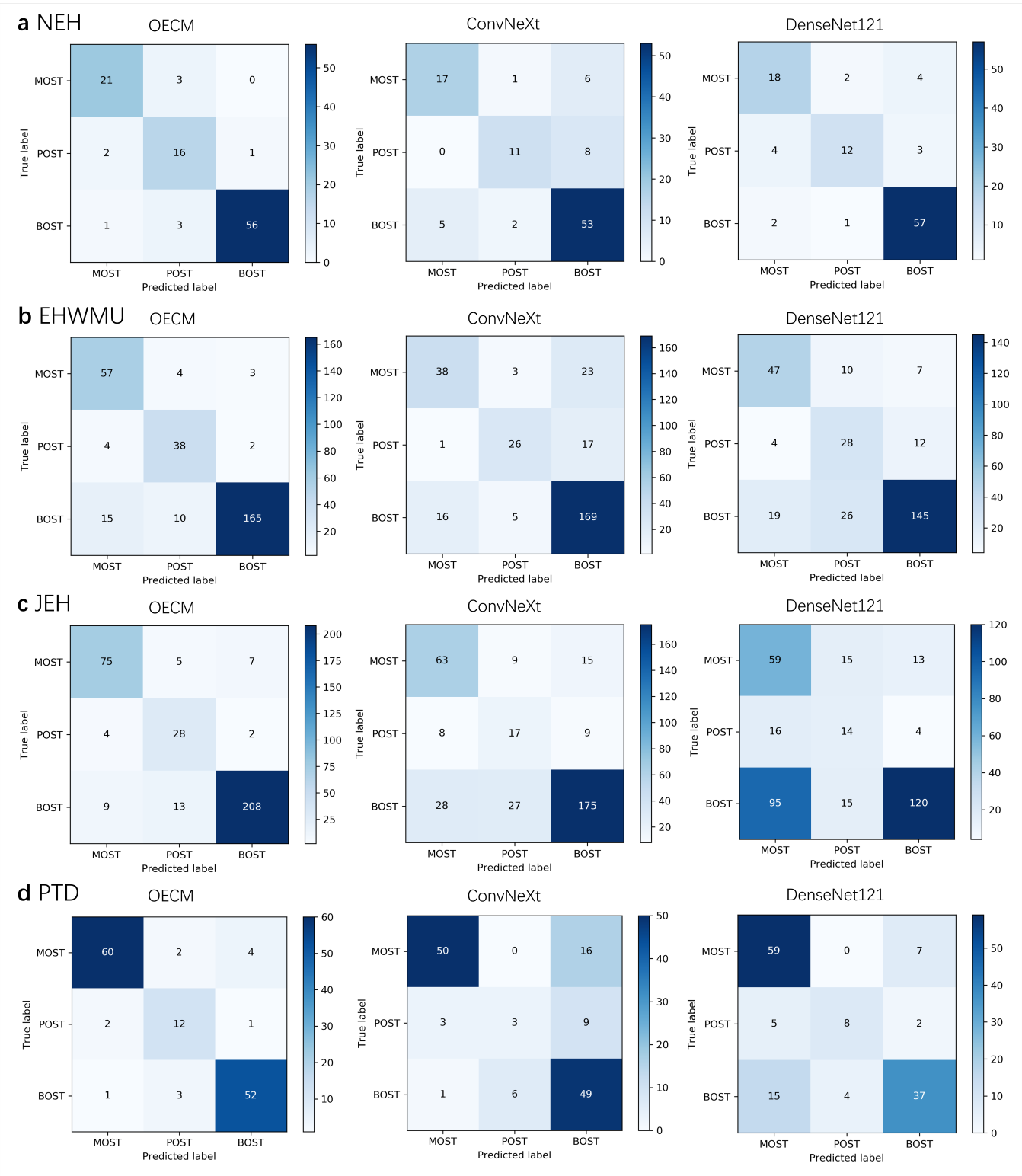


**Fig. S6. Confusion matrices of OECM and CNN models in detecting malignant, premalignant, and benign OSTs.** **a** Internal test. Models are internally evaluated on OST images captured by slit-lamp imaging at NEH. **b** External test. Models are externally evaluated on OST images captured by slit-lamp imaging at EHWMU. **c** External test. Models are externally evaluated on OST images captured by common digital cameras at JEH. **d** Prospective test. Models are prospectively evaluated on OST images captured by slit-lamp imaging at NEH. NEH, Ningbo Eye Hospital. EHWMU, Eye Hospital of Wenzhou Medical University. JEH, Jiangdong Eye Hospital. PTD, prospective test dataset. MOST, malignant ocular surface tumor. POST, premalignant ocular surface tumor. BOST, benign ocular surface tumor. OECM, OSPM-enhanced classification model.


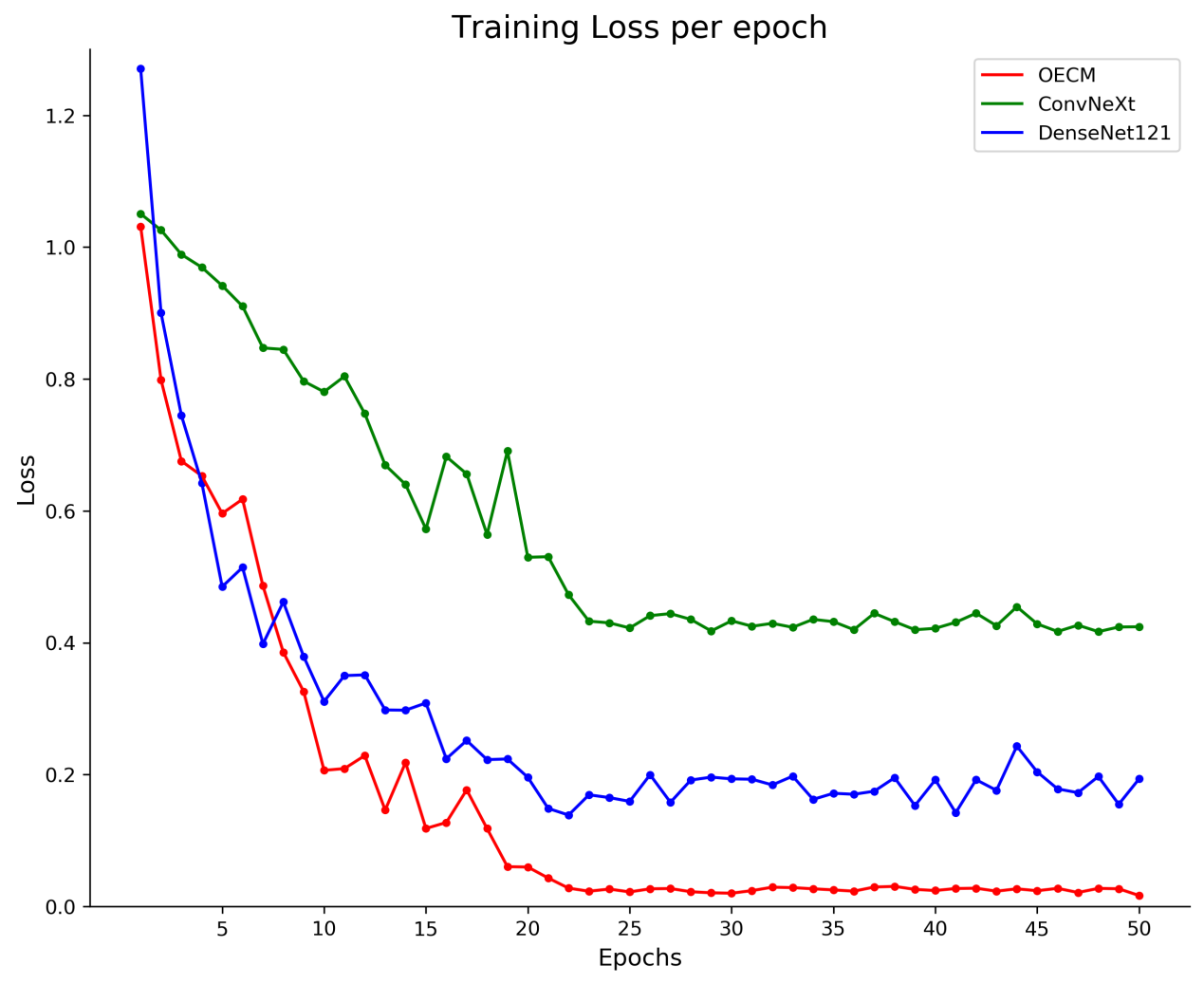


**Fig. S7.** **Loss curves of different deep learning models during training.** The lines depict the training loss over 50 epochs for the different deep learning models. OECM, OSPM-enhanced classification model.

**
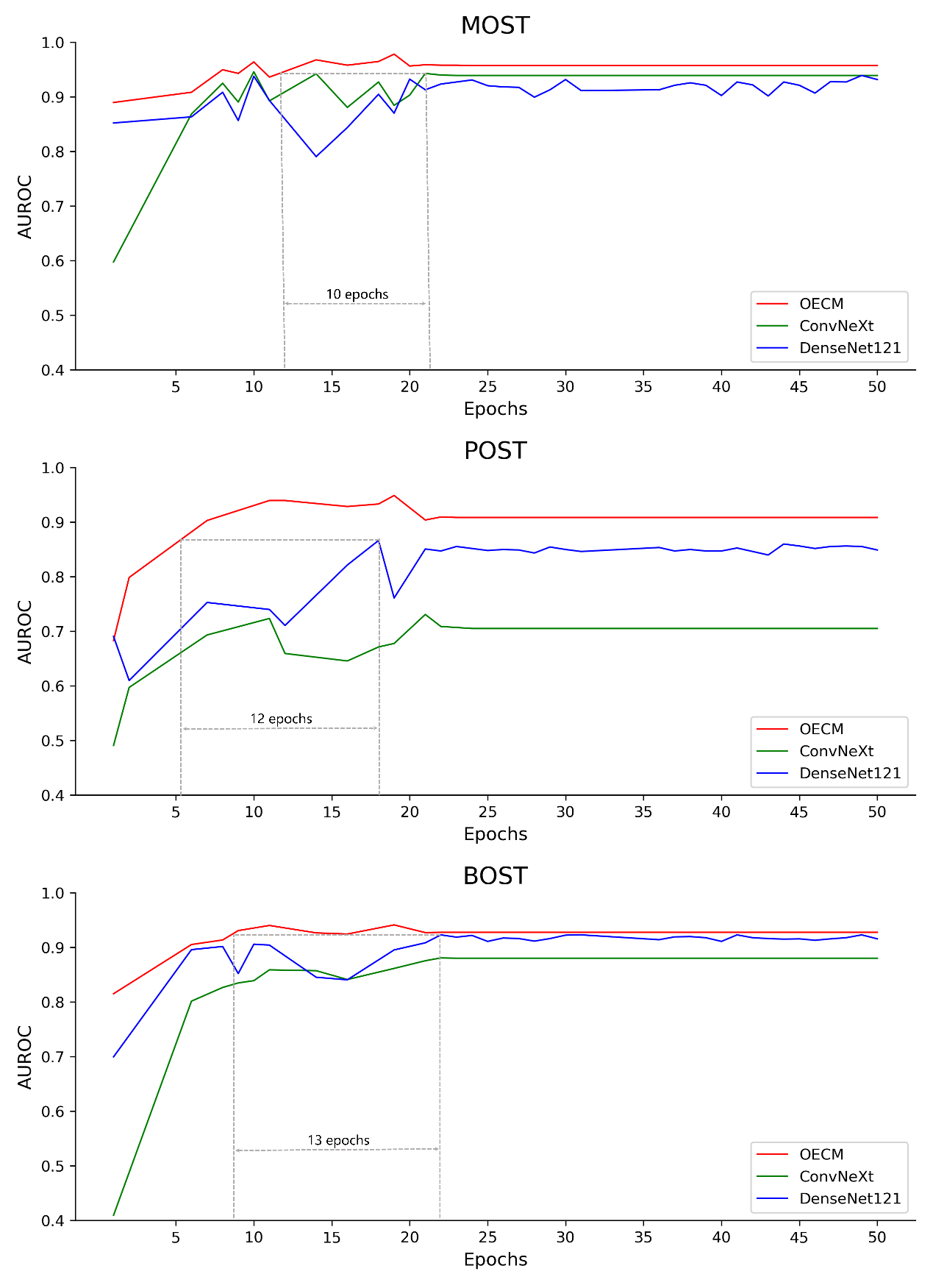
**

**Fig. S8. Training efficiency of OECM and CNN models in detecting malignant, premalignant, and benign OSTs.** Training efficiency denotes the epochs needed to reach training convergence. The performance of the models on the validation set is displayed with identical hyperparameters. The dashed grey lines indicate the epochs when the model checkpoint is saved and the difference in the number of epochs between OECM and the most competitive comparison CNN model is calculated. AUROC, area under the receiver operating characteristic. MOST, malignant ocular surface tumor. POST, premalignant ocular surface tumor. BOST, benign ocular surface tumor. OECM, OSPM-enhanced classification model.

**
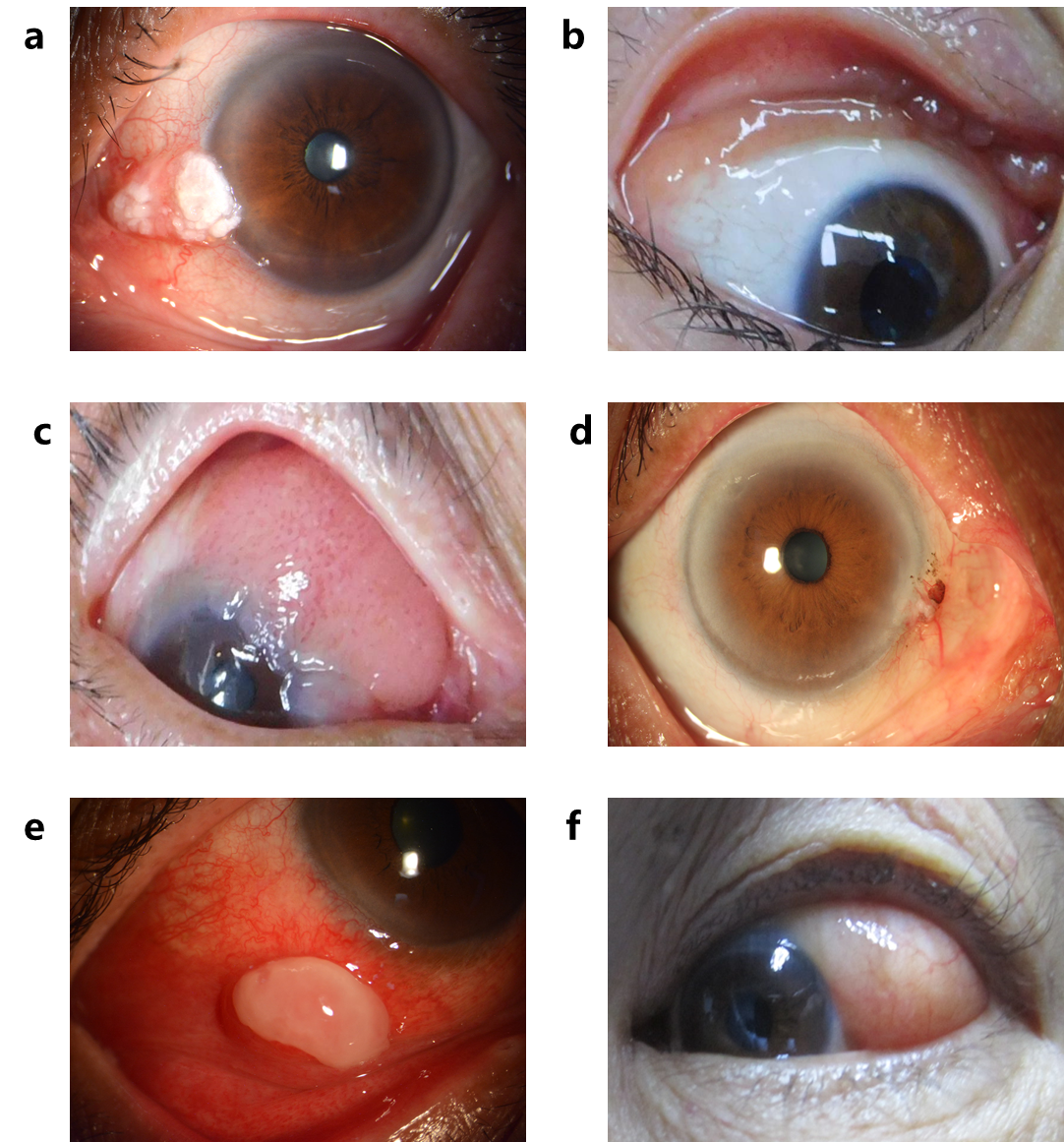
**

**Fig. S9. Typical examples of incorrectly classified images by OECM.** **a** The malignant OST (carcinoma in situ) is misclassified as the premalignant OST. **b** The malignant OST (conjunctival lymphoma) is misclassified as the benign OST. **c** The premalignant OST (conjunctival intraepithelial neoplasia) is misclassified as the malignant OST. **d** The premalignant OST (conjunctival intraepithelial neoplasia) is misclassified as the benign OST. **e** The benign OST (pyogenic granuloma) is misclassified as the malignant OST. **f** The benign OST (lipoma) is misclassified as the premalignant OST. OECM, OSPM-enhanced classification model. OST, ocular surface tumor.

**
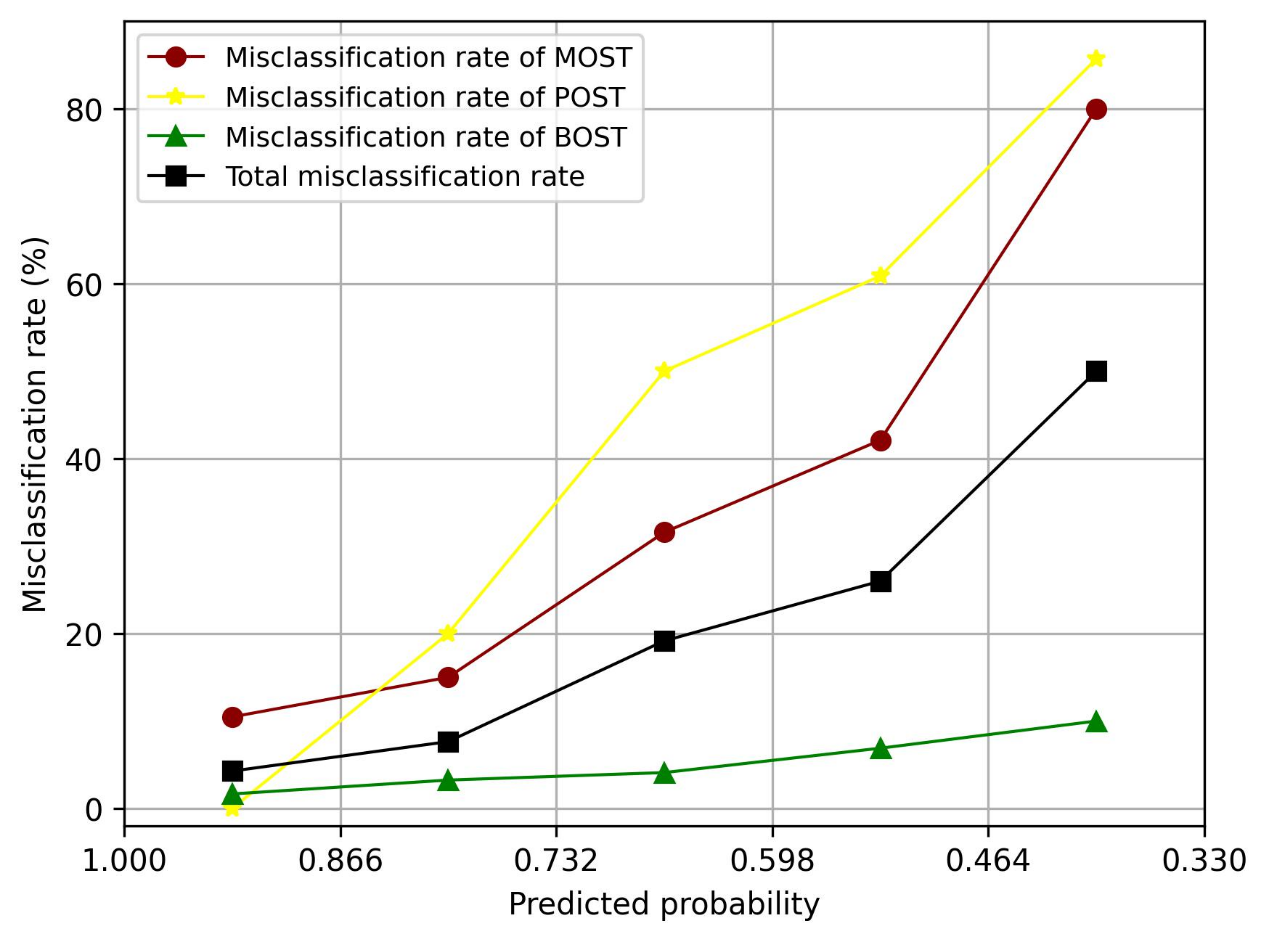
**

**Fig. S10. Correlation between OECM predicted probabilities and misclassification rates.** The misclassification rate denotes the proportion of incorrectly classified images within each predicted probability interval defined by the threshold values. MOST, malignant ocular surface tumor. POST, premalignant ocular surface tumor. BOST, benign ocular surface tumor. OECM, OSPM-enhanced classification model.

**
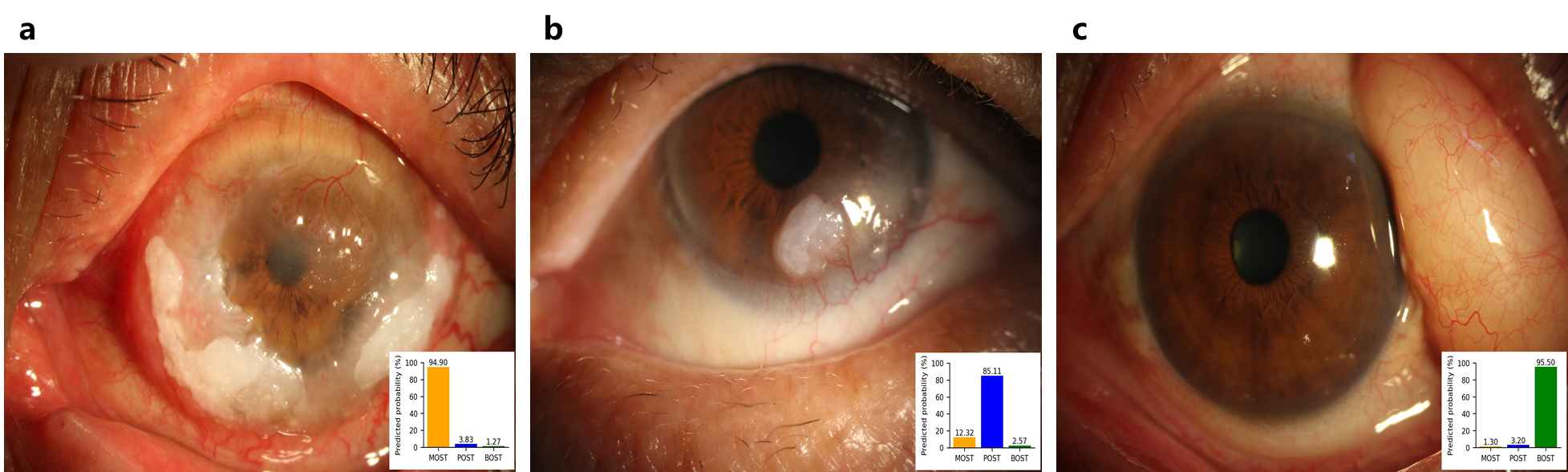
**

**Supplementary Figure. 11. Typical examples of images with predicted probabilities generated by OECM.** The predicted probability map for each type of ocular surface tumor is situated in the lower right corner of the image. **a** MOST (squamous cell carcinoma). **b** POST (conjunctival intraepithelial neoplasia). **c** BOST (lipoma). OECM, OSPM-enhanced classification model. MOST, malignant ocular surface tumor. POST, premalignant ocular surface tumor. BOST, benign ocular surface tumor.


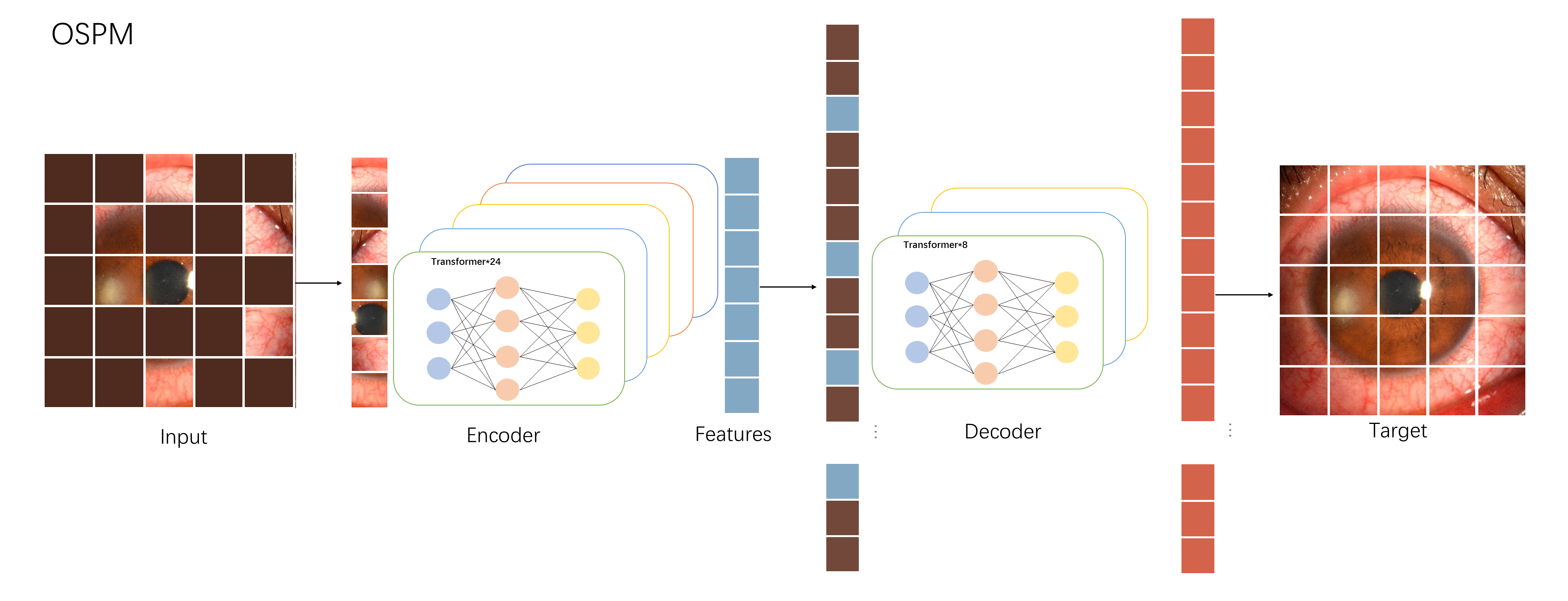


**Fig. S12.** **Architecture of OSPM.** During the pre-training phase, a large random subset of image patches (75%) is masked. The encoder employs a large Vision Transformer (ViT-large) consisting of 24 Transformer blocks and applies it to the smaller subset of visible patches to conserve computational resources. Mask tokens are introduced after the encoder, and the complete set of encoded patches, along with the mask tokens, is processed by a decoder that reconstructs the original image at the pixel level. This decoder is a small Vision Transformer (ViT-small) consisting of 8 Transformer blocks.


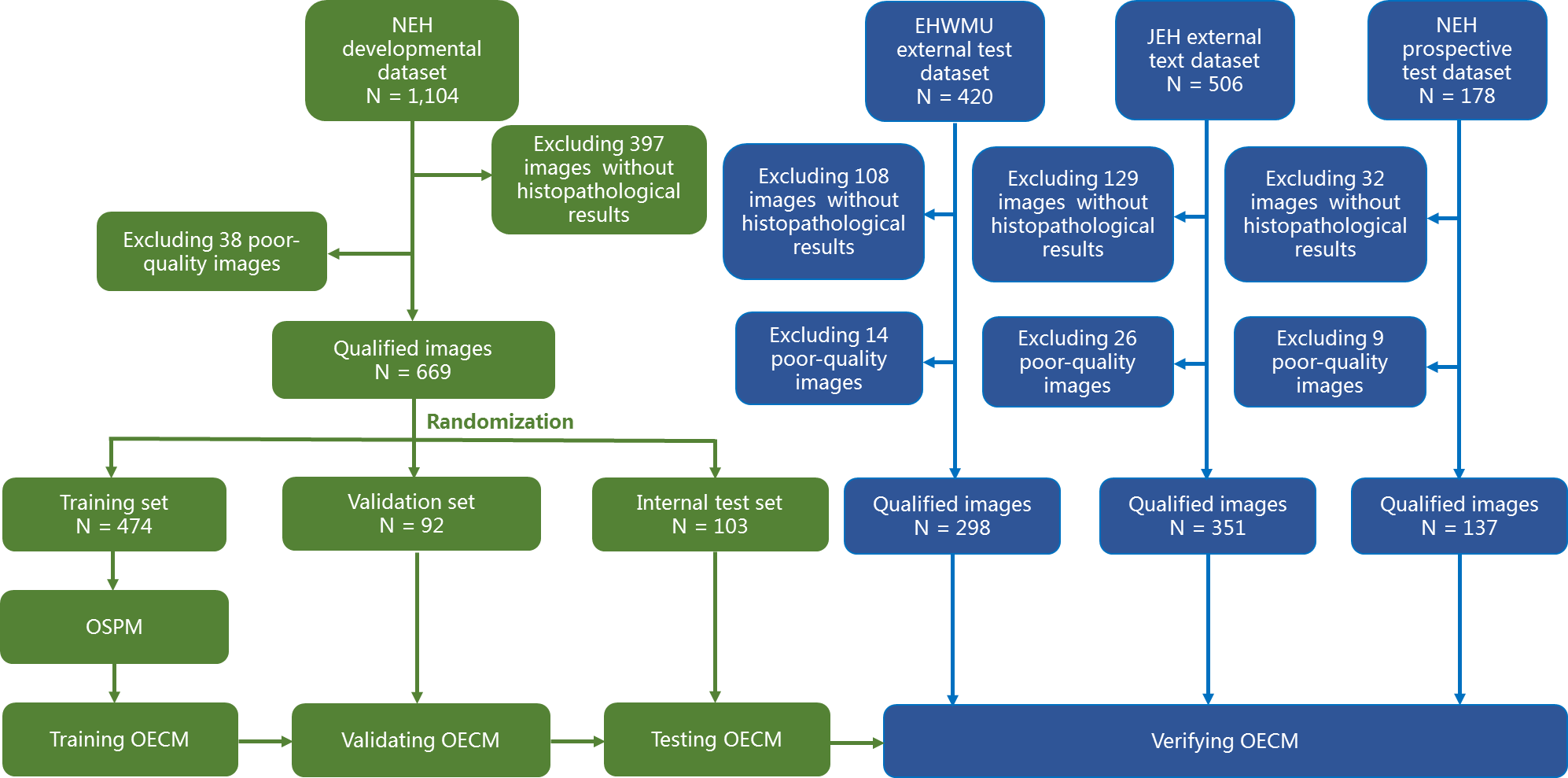


**Fig. S13. Flow diagram of the development and evaluation of OECM.** NEH, Ningbo Eye Hospital. EHWMU, Eye Hospital of Wenzhou Medical University. JEH, Jiangdong Eye Hospital. OSPM, ocular surface pretrained model. OECM, OSPM-enhanced classification model.


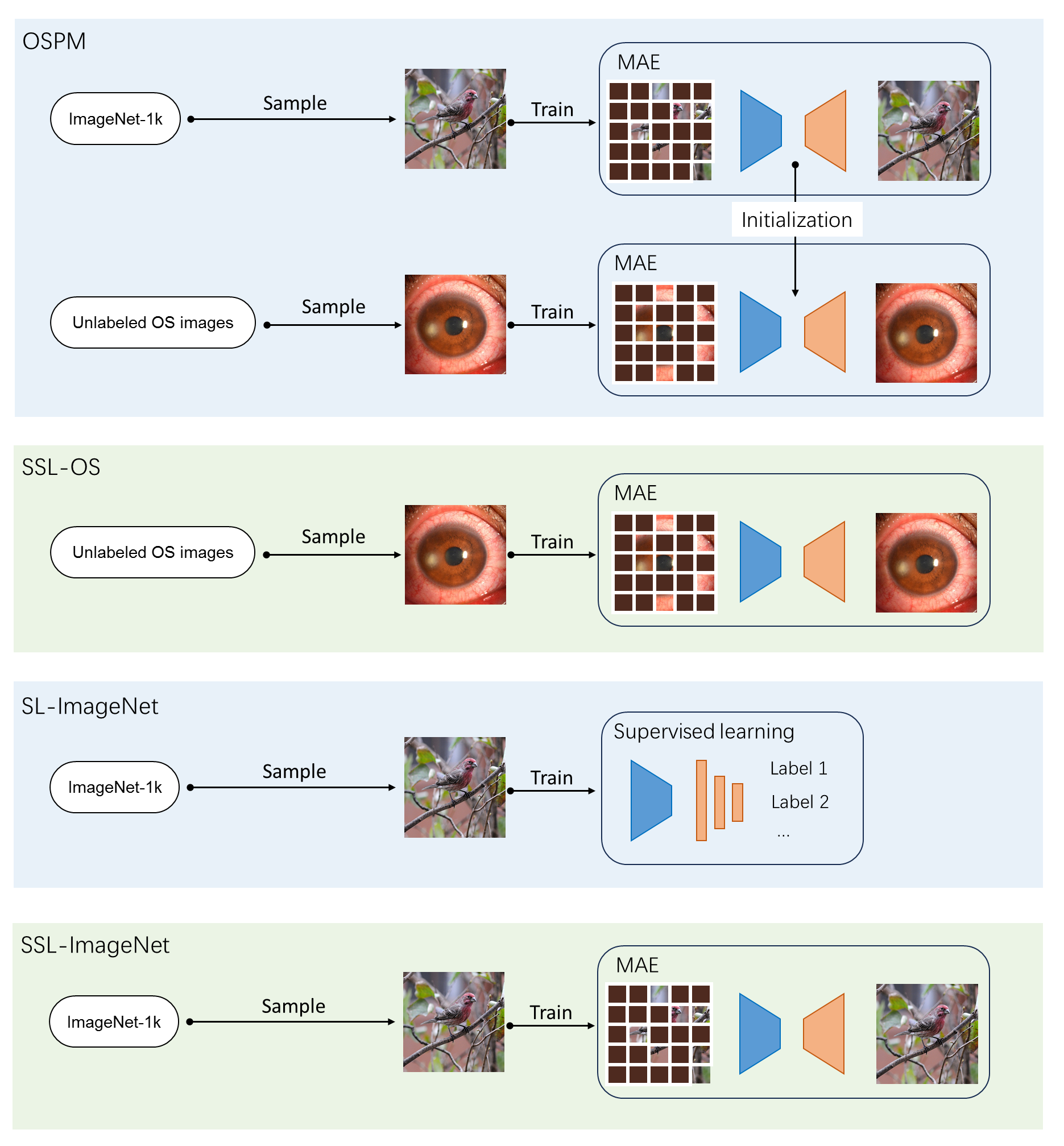


**Fig. S14.** **Diagram of training pipelines of OSPM and other pretrained models.** OSPM trains the model using a masked autoencoder (MAE), first on ImageNet-1k (1.3 million unlabeled natural images) and then on 0.76 million unlabeled ocular surface images; SSL-OS trains the model on ocular surface images (0.76 million unlabeled images) via MAE from scratch; SL-ImageNet trains the model via supervised learning on ImageNet-1k (1.3 million natural images with categorical labels); SSL-ImageNet trains the model on ImageNet-1k (1.3 million unlabeled natural images) via MAE. OSPM, ocular surface pretrained model. SSL, Self-supervised learning. SL, supervised learning.

**Table S1. Summary of datasets used for OSPM development.**

| **Clinical center** | **Location (City)** | **No of Images** | **Device Brand** | **Model** | **File type** | **File size per image** |
| --- | --- | --- | --- | --- | --- | --- |
| NEH | Ningbo | 288,891 | Canton Optics | LS-7 | JPG | 0.11 to 6.42 MB |
| EHWMU | Wenzhou | 208,548 | Sanyo | VPC-MZ3GX | JPG and BMP | 1.36 to 7.21 MB |
| WCSUH | Chengdu | 35,100 | Kanghua | SLM-A | JPG | 0.22 to 6.22 MB |
| FAHHMU | Haerbing | 10,153 | Kanghua | SLM-2 | BMP | 0.89 to 5.51 MB |
| DEH | Daqing | 67,429 | Canon | EOS-450D | JPG | 1.54 to 5.95 MB |
| JEH | Ningbo | 52,620 | Kanghua | SLM-3 | PNG | 2.15 to 3.60 MB |
| AHCU | Chengdu | 19,247 | Kanghua | SLM-B | JPG | 2.80 to 7.72 MB |
| NOC | Ningbo | 57,932 | Nikon | D5200 | JPG | 0.29- to 1.92 MB |
| AHGMU | Guiyang | 9,680 | Kanghua | SLM-KD4 | JPG | 0.31 to 10.40 MB |
| ACH | Xi’an | 6,477 | Canon | EOS-600D | JPG | 0.40 to 2.43 MB |

OSPM, ocular surface pretrained model. NEH, Ningbo Eye Hospital. EHWMU, Eye Hospital of Wenzhou Medical University. WCSUH, West China Second University Hospital. FAHHMU, First Affiliated Hospital of Harbin Medical University. DEH, Daqing Eye Hospital. JEH, Jiangdong Eye Hospital. AHCU, Affiliated Hospital of Chengdu University. NOC, Ningbo Ophthalmic Center. AHGMU, Affiliated Hospital of Guizhou Medical University. ACH, Ankang Center Hospital (Xi’an Medical College). MB, megabyte.

**Table S2. Characteristics of datasets for OECM development and evaluation.**

| Item | NEH dataset | | | EHWMU dataset | JEH dataset | NEH dataset |
| --- | --- | --- | --- | --- | --- | --- |
| Usage | Model development | | | Model external test | Model external test | Model prospective test |
| Total no. of images |  | 669 |  | 298 | 351 | 137 |
| Imaging device |  | Slit-lamp imaging |  | Slit-lamp imaging | Common digital cameras | Slit-lamp imaging |
| Camera model |  | Canton Optics LS-7 |  | Sanyo VPC-MZ3GX | FUJIFILM F450 and NIKON S7000 | Canton Optics LS-7 |
|  | Training set | Validation set | Internal test set |  |  |  |
| Malignant OSTs^a^ | 87/474 (18.4) | 18/92 (19.6) | 24/103 (23.3) | 64/298 (21.5) | 87/351 (24.8) | 66/137 (48.2) |
| Premalignant OSTs^a^ | 69/474 (14.6) | 14/92 (15.2) | 19/103 (18.4) | 44/298 (14.8) | 34/351 (9.7) | 15/137 (10.9) |
| Benign OSTs^a^ | 318/474 (67.0) | 60/92 (65.2) | 60/103 (58.3) | 190/298 (63.7) | 230/351 (65.5) | 56/137 (40.9) |

^a^Data are no. of images/total no. (%) unless otherwise indicated. OECM, OSPM-enhanced classification model. NEH, Ningbo Eye Hospital. EHWMU, Eye Hospital of Wenzhou Medical University. JEH, Jiangdong Eye Hospital. OST, ocular surface tumor.

**Table S3. Performance of models using different pretraining approaches in the NEH internal test dataset.**

| **One-vs.-rest classification** | **NEH internal test dataset** | | | | |
| --- | --- | --- | --- | --- | --- |
|  | **Accuracy (95% CI)** | **Sensitivity (95% CI)** | **Specificity (95% CI)** | **AUROC (95% CI)** | **AUPRC (95% CI)** |
| **MOST vs. POST + BOST** | | | | | |
| OSPM | 94.17 (89.32, 98.06) | 87.50 (73.08, 100.00) | 96.20 (91.36, 100.00) | 0.986 (0.967, 0.998) | 0.964 (0.908, 0.995) |
| SSL-OS | 95.15 (91.26, 99.03) | 83.33 (68.18, 96.00) | 98.73 (96.00, 100.00) | 0.989 (0.971, 0.999) | 0.971 (0.922, 0.998) |
| SL-ImageNet | 92.23 (86.41, 97.09) | 83.33 (66.67, 96.30) | 94.94 (89.63, 98.79) | 0.980 (0.954, 0.996) | 0.937 (0.851, 0.988) |
| SSL-ImageNet | 87.38 (80.58, 93.20) | 70.83 (52.27, 88.69) | 92.41 (86.06, 97.50) | 0.939 (0.888, 0.978) | 0.814 (0.650, 0.940) |
| **POST vs. MOST + BOST** | | | | | |
| OSPM | 91.26 (85.44, 96.12) | 84.21 (66.67, 100.00) | 92.86 (87.01, 97.70) | 0.977 (0.951, 0.996) | 0.923 (0.823, 0.987) |
| SSL-OS | 93.20 (88.35, 98.06) | 100.00 (100.00, 100.00) | 91.67 (85.71, 97.56) | 0.976 (0.948, 0.995) | 0.899 (0.778, 0.982) |
| SL-ImageNet | 89.32 (82.52, 95.15) | 68.42 (45.63, 88.89) | 94.05 (88.51, 98.81) | 0.962 (0.922, 0.990) | 0.876 (0.736, 0.963) |
| SSL-ImageNet | 90.29 (84.47, 95.15) | 78.95 (59.09, 95.12) | 92.86 (87.27, 97.67) | 0.944 (0.883, 0.990) | 0.859 (0.719, 0.959) |
| **BOST vs. MOST + POST** | | | | | |
| OSPM | 95.15 (90.29, 99.03) | 93.33 (86.73, 98.45) | 97.67 (92.50, 100.00) | 0.993 (0.980, 1.000) | 0.995 (0.985, 1.000) |
| SSL-OS | 94.17 (89.32, 98.06) | 91.67 (84.13, 98.28) | 97.67 (92.11, 100.00) | 0.990 (0.975, 0.999) | 0.993 (0.983, 0.999) |
| SL-ImageNet | 95.15 (90.29, 99.03) | 96.67 (91.38, 100.00) | 93.02 (84.62, 100.00) | 0.988 (0.970, 0.999) | 0.992 (0.980, 0.999) |
| SSL-ImageNet | 89.32 (82.52, 94.17) | 90.00 (82.46, 96.67) | 88.37 (78.57, 97.18) | 0.945 (0.886, 0.989) | 0.947 (0.877, 0.993) |

NEH, Ningbo Eye Hospital. AUROC, area under the receiver operating characteristic. AUPRC, area under the precision-recall curve. CI, confidence interval. MOST, malignant ocular surface tumor. POST, premalignant ocular surface tumor. BOST, benign ocular surface tumor. OSPM, ocular surface pretrained model. OSPM trains the model using a masked autoencoder (MAE), first on ImageNet-1k (1.3 million unlabeled natural images) and then on 0.76 million unlabeled ocular surface images. SSL-OS trains the model on ocular surface images (0.76 million unlabeled images) via MAE from scratch. SL-ImageNet trains the model via supervised learning on ImageNet-1k (1.3 million natural images with categorical labels). SSL-ImageNet trains the model on ImageNet-1k (1.3 million unlabeled natural images) via MAE.

**Table S4. Performance of models using different pretraining approaches in the** **EHWMU external test dataset.**

| **One-vs.-rest classification** | **EHWMU external test dataset** | | | | |
| --- | --- | --- | --- | --- | --- |
|  | **Accuracy (95% CI)** | **Sensitivity (95% CI)** | **Specificity (95% CI)** | **AUROC (95% CI)** | **AUPRC (95% CI)** |
| **MOST vs. POST + BOST** | | | | | |
| OSPM | 91.28 (87.92, 94.30) | 89.06 (80.37, 96.04) | 91.88 (88.36, 95.11) | 0.959 (0.931, 0.981) | 0.847 (0.744, 0.934) |
| SSL-OS | 89.93 (86.58, 93.29) | 82.81 (72.73, 91.48) | 91.88 (88.56, 95.21) | 0.960 (0.938, 0.978) | 0.832 (0.735, 0.921) |
| SL-ImageNet | 88.93 (85.23, 92.62) | 78.12 (67.85, 87.50) | 91.88 (88.23, 95.38) | 0.959 (0.929, 0.980) | 0.869 (0.775, 0.942) |
| SSL-ImageNet | 82.55 (78.19, 86.75) | 53.12 (40.74, 64.97) | 90.60 (86.76, 94.12) | 0.877 (0.831, 0.918) | 0.686 (0.571, 0.788) |
| **POST vs. MOST + BOST** | | | | | |
| OSPM | 93.29 (90.27, 95.97) | 86.36 (75.76, 95.74) | 94.49 (91.46, 97.20) | 0.960 (0.919, 0.991) | 0.913 (0.843, 0.969) |
| SSL-OS | 92.28 (89.26, 95.30) | 86.36 (76.04, 95.45) | 93.31 (90.24, 96.37) | 0.974 (0.949, 0.993) | 0.920 (0.858, 0.970) |
| SL-ImageNet | 89.60 (85.91, 92.95) | 68.18 (54.55, 81.58) | 93.31 (90.08, 96.32) | 0.936 (0.893, 0.970) | 0.790 (0.679, 0.880) |
| SSL-ImageNet | 77.52 (72.82, 82.21) | 59.09 (44.17, 73.33) | 80.71 (75.82, 85.29) | 0.820 (0.753, 0.885) | 0.525 (0.378, 0.667) |
| **BOST vs. MOST + POST** | | | | | |
| OSPM | 89.93 (86.24, 93.29) | 86.84 (81.94, 91.51) | 95.37 (90.94, 99.06) | 0.957 (0.928, 0.980) | 0.968 (0.941, 0.989) |
| SSL-OS | 88.93 (85.23, 92.28) | 86.32 (81.23, 90.98) | 93.52 (88.65, 98.04) | 0.963 (0.942, 0.982) | 0.980 (0.966, 0.991) |
| SL-ImageNet | 85.23 (80.87, 89.26) | 86.32 (81.35, 90.80) | 83.33 (76.21, 90.05) | 0.946 (0.918, 0.968) | 0.969 (0.950, 0.984) |
| SSL-ImageNet | 73.49 (68.46, 78.19) | 73.16 (66.58, 79.08) | 74.07 (65.28, 81.65) | 0.835 (0.783, 0.880) | 0.875 (0.822, 0.927) |

EHWMU, Eye Hospital of Wenzhou Medical University. AUROC, area under the receiver operating characteristic. AUPRC, area under the precision-recall curve. CI, confidence interval. MOST, malignant ocular surface tumor. POST, premalignant ocular surface tumor. BOST, benign ocular surface tumor. OSPM, ocular surface pretrained model. OSPM trains the model using a masked autoencoder (MAE), first on ImageNet-1k (1.3 million unlabeled natural images) and then on 0.76 million unlabeled ocular surface images. SSL-OS trains the model on ocular surface images (0.76 million unlabeled images) via MAE from scratch. SL-ImageNet trains the model via supervised learning on ImageNet-1k (1.3 million natural images with categorical labels). SSL-ImageNet trains the model on ImageNet-1k (1.3 million unlabeled natural images) via MAE.

**Table S5. Performance of models using different pretraining approaches in the JEH external test dataset.**

| **One-vs.-rest classification** | **JEH external test dataset** | | | | |
| --- | --- | --- | --- | --- | --- |
|  | **Accuracy (95% CI)** | **Sensitivity (95% CI)** | **Specificity (95% CI)** | **AUROC (95% CI)** | **AUPRC (95% CI)** |
| **MOST vs. POST + BOST** | | | | | |
| OSPM | 92.88 (90.03, 95.44) | 86.21 (78.69, 93.29) | 95.08 (92.47, 97.53) | 0.927 (0.892, 0.959) | 0.817 (0.729, 0.897) |
| SSL-OS | 85.47 (82.05, 89.17) | 79.31 (71.04, 87.80) | 87.50 (83.64, 91.40) | 0.899 (0.863, 0.936) | 0.759 (0.668, 0.846) |
| SL-ImageNet | 77.49 (72.93, 81.77) | 73.56 (63.64, 82.58) | 78.79 (73.86, 83.77) | 0.837 (0.782, 0.885) | 0.672 (0.570, 0.770) |
| SSL-ImageNet | 66.38 (61.54, 71.23) | 68.97 (59.40, 78.79) | 65.53 (59.77, 71.22) | 0.738 (0.680, 0.795) | 0.454 (0.363, 0.568) |
| **POST vs. MOST + BOST** | | | | | |
| OSPM | 93.16 (90.31, 95.73) | 82.35 (68.00, 94.59) | 94.32 (91.57, 96.82) | 0.891 (0.797, 0.963) | 0.744 (0.595, 0.871) |
| SSL-OS | 90.60 (87.18, 93.73) | 50.00 (33.33, 66.67) | 94.95 (92.48, 97.20) | 0.844 (0.753, 0.921) | 0.570 (0.410, 0.722) |
| SL-ImageNet | 93.16 (90.31, 95.73) | 50.00 (33.33, 67.57) | 97.79 (96.18, 99.36) | 0.838 (0.741, 0.920) | 0.614 (0.436, 0.768) |
| SSL-ImageNet | 80.34 (76.07, 84.33) | 61.76 (45.16, 78.57) | 82.33 (77.78, 86.52) | 0.823 (0.741, 0.898) | 0.404 (0.259, 0.573) |
| **BOST vs. MOST + POST** | | | | | |
| OSPM | 91.17 (88.03, 94.02) | 90.43 (86.62, 94.21) | 92.56 (87.44, 96.85) | 0.940 (0.910, 0.965) | 0.964 (0.941, 0.982) |
| SSL-OS | 88.03 (84.75, 91.45) | 87.83 (83.71, 91.88) | 88.43 (82.68, 93.80) | 0.925 (0.892, 0.954) | 0.952 (0.926, 0.975) |
| SL-ImageNet | 76.35 (71.93, 80.91) | 76.96 (71.59, 82.64) | 75.21 (67.21, 82.74) | 0.837 (0.788, 0.881) | 0.881 (0.827, 0.928) |
| SSL-ImageNet | 59.26 (54.13, 64.10) | 45.65 (39.33, 52.00) | 85.12 (78.84, 91.04) | 0.763 (0.708, 0.813) | 0.845 (0.792, 0.891) |

JEH, Jiangdong Eye Hospital. AUROC, area under the receiver operating characteristic. AUPRC, area under the precision-recall curve. CI, confidence interval. MOST, malignant ocular surface tumor. POST, premalignant ocular surface tumor. BOST, benign ocular surface tumor. OSPM, ocular surface pretrained model. OSPM trains the model using a masked autoencoder (MAE), first on ImageNet-1k (1.3 million unlabeled natural images) and then on 0.76 million unlabeled ocular surface images. SSL-OS trains the model on ocular surface images (0.76 million unlabeled images) via MAE from scratch. SL-ImageNet trains the model via supervised learning on ImageNet-1k (1.3 million natural images with categorical labels). SSL-ImageNet trains the model on ImageNet-1k (1.3 million unlabeled natural images) via MAE.

**Table S6. Performance of models using different pretraining approaches in the prospective test dataset.**

| **One-vs.-rest classification** | **Prospective test dataset** | | | | |
| --- | --- | --- | --- | --- | --- |
|  | **Accuracy (95% CI)** | **Sensitivity (95% CI)** | **Specificity (95% CI)** | **AUROC (95% CI)** | **AUPRC (95% CI)** |
| **MOST vs. POST + BOST** | | | | | |
| OSPM | 93.43 (89.40, 97.08) | 90.91 (83.82, 97.01) | 95.77 (90.78, 100.00) | 0.945 (0.898, 0.982) | 0.920 (0.841, 0.985) |
| SSL-OS | 83.94 (78.10, 89.78) | 75.76 (65.57, 85.71) | 91.55 (85.04, 97.33) | 0.912 (0.857, 0.959) | 0.882 (0.793, 0.963) |
| SL-ImageNet | 83.21 (77.37, 89.05) | 84.85 (75.96, 92.97) | 81.69 (73.07, 90.00) | 0.907 (0.851, 0.949) | 0.912 (0.853, 0.954) |
| SSL-ImageNet | 75.18 (67.88, 82.48) | 78.79 (68.29, 88.71) | 71.83 (61.67, 82.52) | 0.836 (0.764, 0.902) | 0.816 (0.720, 0.913) |
| **POST vs. MOST + BOST** | | | | | |
| OSPM | 94.16 (89.78, 97.81) | 80.00 (57.14, 100.00) | 95.90 (91.77, 99.17) | 0.887 (0.749, 0.990) | 0.730 (0.485, 0.921) |
| SSL-OS | 92.70 (87.59, 96.35) | 73.33 (50.00, 93.55) | 95.08 (90.55, 98.37) | 0.902 (0.777, 0.983) | 0.716 (0.484, 0.896) |
| SL-ImageNet | 91.24 (86.13, 95.62) | 40.00 (15.38, 66.67) | 97.54 (94.33, 100.00) | 0.872 (0.779, 0.944) | 0.596 (0.356, 0.814) |
| SSL-ImageNet | 88.32 (82.48, 93.43) | 60.00 (33.33, 84.62) | 91.80 (86.42, 96.31) | 0.831 (0.675, 0.950) | 0.464 (0.266, 0.746) |
| **BOST vs. MOST + POST** | | | | | |
| OSPM | 93.43 (89.05, 97.08) | 92.86 (85.45, 98.37) | 93.83 (88.46, 98.70) | 0.965 (0.931, 0.990) | 0.947 (0.885, 0.989) |
| SSL-OS | 81.02 (74.45, 86.86) | 83.93 (74.07, 92.80) | 79.01 (70.12, 87.57) | 0.897 (0.838, 0.945) | 0.817 (0.709, 0.930) |
| SL-ImageNet | 80.29 (73.72, 86.86) | 78.57 (67.35, 88.89) | 81.48 (72.97, 89.57) | 0.874 (0.810, 0.928) | 0.801 (0.696, 0.891) |
| SSL-ImageNet | 70.80 (62.77, 78.10) | 55.36 (42.57, 68.69) | 81.48 (72.67, 89.47) | 0.782 (0.706, 0.854) | 0.666 (0.547, 0.805) |

AUROC, area under the receiver operating characteristic. AUPRC, area under the precision-recall curve. CI, confidence interval. MOST, malignant ocular surface tumor. POST, premalignant ocular surface tumor. BOST, benign ocular surface tumor. OSPM, ocular surface pretrained model. OSPM trains the model using a masked autoencoder (MAE), first on ImageNet-1k (1.3 million unlabeled natural images) and then on 0.76 million unlabeled ocular surface images. SSL-OS trains the model on ocular surface images (0.76 million unlabeled images) via MAE from scratch. SL-ImageNet trains the model via supervised learning on ImageNet-1k (1.3 million natural images with categorical labels). SSL-ImageNet trains the model on ImageNet-1k (1.3 million unlabeled natural images) via MAE.

**Table S7. Performance of models using different SSL approaches in the NEH internal test dataset.**

| **One-vs.-rest classification** | **NEH internal test dataset** | | | | |
| --- | --- | --- | --- | --- | --- |
|  | **Accuracy (95% CI)** | **Sensitivity (95% CI)** | **Specificity (95% CI)** | **AUROC (95% CI)** | **AUPRC (95% CI)** |
| **MOST vs. POST + BOST** | | | | | |
| MAE | 94.17 (89.32, 98.06) | 87.50 (73.08, 100.00) | 96.20 (91.36, 100.00) | 0.986 (0.967, 0.998) | 0.964 (0.908, 0.995) |
| DINO | 85.44 (78.64, 91.77) | 79.17 (61.20, 94.44) | 87.34 (79.62, 94.48) | 0.916 (0.841, 0.970) | 0.807 (0.638, 0.930) |
| EVA | 93.20 (88.35, 98.06) | 79.17 (61.11, 95.00) | 97.47 (93.42, 100.00) | 0.963 (0.903, 0.999) | 0.934 (0.843, 0.996) |
| iBOT | 89.32 (83.50, 95.15) | 91.67 (80.00, 100.00) | 88.61 (81.24, 95.06) | 0.977 (0.946, 0.997) | 0.915 (0.791, 0.991) |
| **POST vs. MOST + BOST** | | | | | |
| MAE | 91.26 (85.44, 96.12) | 84.21 (66.67, 100.00) | 92.86 (87.01, 97.70) | 0.977 (0.951, 0.996) | 0.923 (0.823, 0.987) |
| DINO | 88.35 (82.52, 94.17) | 63.16 (40.00, 84.62) | 94.05 (88.76, 98.74) | 0.948 (0.898, 0.986) | 0.843 (0.693, 0.953) |
| EVA | 92.23 (86.41, 97.09) | 89.47 (72.22, 100.00) | 92.86 (86.59, 97.67) | 0.908 (0.756, 1.000) | 0.907 (0.764, 1.000) |
| iBOT | 91.26 (85.44, 96.12) | 73.68 (53.85, 94.12) | 95.24 (90.59, 98.86) | 0.921 (0.821, 0.986) | 0.834 (0.670, 0.948) |
| **BOST vs. MOST + POST** | | | | | |
| MAE | 95.15 (90.29, 99.03) | 93.33 (86.73, 98.45) | 97.67 (92.50, 100.00) | 0.993 (0.980, 1.000) | 0.995 (0.985, 1.000) |
| DINO | 91.26 (85.44, 96.12) | 90.00 (81.51, 96.77) | 93.02 (84.44, 100.00) | 0.981 (0.959, 0.996) | 0.987 (0.971, 0.997) |
| EVA | 91.26 (85.44, 96.12) | 91.67 (83.82, 98.33) | 90.70 (81.40, 97.96) | 0.989 (0.973, 0.998) | 0.993 (0.981, 0.999) |
| iBOT | 88.35 (81.55, 94.17) | 85.00 (75.35, 93.75) | 93.02 (84.62, 100.00) | 0.989 (0.970, 0.999) | 0.992 (0.978, 1.000) |

Models pretrained with different self-supervised learning (SSL) approaches, including masked autoencoders (MAE), DINO, EVA, and iBOT, undergo the same fine-tuning processes for downstream tasks. The OSPM-enhanced classification model is pretrained with MAE. NEH, Ningbo Eye Hospital. AUROC, area under the receiver operating characteristic. AUPRC, area under the precision-recall curve. CI, confidence interval. MOST, malignant ocular surface tumor. POST, premalignant ocular surface tumor. BOST, benign ocular surface tumor. MAE, Masked Autoencoders. SSL, Self-supervised learning.

**Table S8. Performance of models using different SSL approaches in the EHWMU external test dataset.**

| **One-vs.-rest classification** | **EHWMU external test dataset** | | | | |
| --- | --- | --- | --- | --- | --- |
|  | **Accuracy (95% CI)** | **Sensitivity (95% CI)** | **Specificity (95% CI)** | **AUROC (95% CI)** | **AUPRC (95% CI)** |
| **MOST vs. POST + BOST** | | | | | |
| MAE | 91.28 (87.92, 94.30) | 89.06 (80.37, 96.04) | 91.88 (88.36, 95.11) | 0.959 (0.931, 0.981) | 0.847 (0.744, 0.934) |
| DINO | 90.60 (87.25, 93.62) | 78.12 (67.45, 87.86) | 94.02 (90.70, 96.93) | 0.962 (0.940, 0.979) | 0.841 (0.734, 0.935) |
| EVA | 90.27 (86.91, 93.62) | 68.75 (57.89, 80.28) | 96.15 (93.36, 98.31) | 0.925 (0.881, 0.963) | 0.845 (0.770, 0.905) |
| iBOT | 87.92 (84.23, 91.28) | 84.38 (75.00, 92.73) | 88.89 (84.61, 92.61) | 0.940 (0.910, 0.966) | 0.844 (0.770, 0.905) |
| **POST vs. MOST + BOST** | | | | | |
| MAE | 93.29 (90.27, 95.97) | 86.36 (75.76, 95.74) | 94.49 (91.46, 97.20) | 0.960 (0.919, 0.991) | 0.913 (0.843, 0.969) |
| DINO | 86.58 (82.55, 90.27) | 79.55 (66.67, 91.15) | 87.80 (83.59, 91.54) | 0.929 (0.877, 0.968) | 0.808 (0.708, 0.892) |
| EVA | 91.28 (87.92, 94.30) | 84.09 (72.09, 94.29) | 92.52 (89.20, 95.58) | 0.934 (0.883, 0.977) | 0.863 (0.766, 0.939) |
| iBOT | 86.91 (83.22, 90.60) | 68.18 (53.65, 81.40) | 90.16 (86.59, 93.60) | 0.931 (0.897, 0.960) | 0.704 (0.567, 0.836) |
| **BOST vs. MOST + POST** | | | | | |
| MAE | 89.93 (86.24, 93.29) | 86.84 (81.94, 91.51) | 95.37 (90.94, 99.06) | 0.957 (0.928, 0.980) | 0.968 (0.941, 0.989) |
| DINO | 83.89 (79.87, 87.92) | 81.58 (75.71, 86.84) | 87.96 (81.82, 94.06) | 0.944 (0.918, 0.966) | 0.967 (0.948, 0.981) |
| EVA | 92.28 (89.26, 94.97) | 93.68 (89.84, 96.67) | 89.81 (84.17, 95.00) | 0.963 (0.947, 0.982) | 0.975 (0.944, 0.996) |
| iBOT | 85.57 (81.54, 89.60) | 81.58 (75.79, 86.98) | 92.59 (87.25, 97.27) | 0.962 (0.943, 0.979) | 0.975 (0.969, 0.984) |

Models pretrained with different self-supervised learning (SSL) approaches, including masked autoencoders (MAE), DINO, EVA, and iBOT, undergo the same fine-tuning processes for downstream tasks. The OSPM-enhanced classification model is pretrained with MAE. EHWMU, Eye Hospital of Wenzhou Medical University. AUROC, area under the receiver operating characteristic. AUPRC, area under the precision-recall curve. CI, confidence interval. MOST, malignant ocular surface tumor. POST, premalignant ocular surface tumor. BOST, benign ocular surface tumor.

**Table S9. Performance of models using different SSL approaches in the JEH external test dataset.**

| **One-vs.-rest classification** | **JEH external test dataset** | | | | |
| --- | --- | --- | --- | --- | --- |
|  | **Accuracy (95% CI)** | **Sensitivity (95% CI)** | **Specificity (95% CI)** | **AUROC (95% CI)** | **AUPRC (95% CI)** |
| **MOST vs. POST + BOST** | | | | | |
| MAE | 92.88 (90.03, 95.44) | 86.21 (78.69, 93.29) | 95.08 (92.47, 97.53) | 0.927 (0.892, 0.959) | 0.817 (0.729, 0.897) |
| DINO | 53.85 (48.43, 58.69) | 83.91 (75.88, 91.57) | 43.94 (37.64, 49.62) | 0.715 (0.647, 0.776) | 0.476 (0.369, 0.582) |
| EVA | 74.64 (70.09, 79.20) | 89.66 (82.35, 95.51) | 69.70 (64.18, 75.10) | 0.887 (0.841, 0.927) | 0.738 (0.644, 0.838) |
| iBOT | 70.37 (65.24, 75.21) | 93.10 (87.18, 97.83) | 62.88 (57.03, 68.52) | 0.880 (0.834, 0.920) | 0.729 (0.622, 0.833) |
| **POST vs. MOST + BOST** | | | | | |
| MAE | 93.16 (90.31, 95.73) | 82.35 (68.00, 94.59) | 94.32 (91.57, 96.82) | 0.891 (0.797, 0.963) | 0.744 (0.595, 0.871) |
| DINO | 89.46 (86.32, 92.59) | 38.24 (21.80, 54.84) | 94.95 (92.50, 97.24) | 0.814 (0.711, 0.898) | 0.478 (0.316, 0.644) |
| EVA | 88.60 (85.19, 91.74) | 73.53 (58.33, 87.88) | 90.22 (86.85, 93.38) | 0.855 (0.755, 0.940) | 0.738 (0.594, 0.870) |
| iBOT | 86.04 (82.34, 89.47) | 64.71 (48.14, 80.00) | 88.33 (84.71, 91.59) | 0.811 (0.710, 0.897) | 0.607 (0.432, 0.760) |
| **BOST vs. MOST + POST** | | | | | |
| MAE | 91.17 (88.03, 94.02) | 90.43 (86.62, 94.21) | 92.56 (87.44, 96.85) | 0.940 (0.910, 0.965) | 0.964 (0.941, 0.982) |
| DINO | 54.13 (48.72, 59.26) | 36.96 (30.48, 43.29) | 86.78 (80.57, 92.59) | 0.740 (0.683, 0.794) | 0.816 (0.754, 0.875) |
| EVA | 70.66 (65.80, 75.50) | 57.39 (50.82, 63.68) | 95.87 (91.87, 99.17) | 0.899 (0.865, 0.931) | 0.936 (0.898, 0.966) |
| iBOT | 63.82 (58.69, 69.23) | 46.96 (40.18, 53.53) | 95.87 (92.12, 99.13) | 0.899 (0.860, 0.931) | 0.936 (0.902, 0.962) |

Models pretrained with different self-supervised learning (SSL) approaches, including masked autoencoders (MAE), DINO, EVA, and iBOT, undergo the same fine-tuning processes for downstream tasks. The OSPM-enhanced classification model is pretrained with MAE. JEH, Jiangdong Eye Hospital. AUROC, area under the receiver operating characteristic. AUPRC, area under the precision-recall curve. CI, confidence interval. MOST, malignant ocular surface tumor. POST, premalignant ocular surface tumor. BOST, benign ocular surface tumor.

**Table S10. Performance of models using different SSL approaches in the prospective test dataset.**

| **One-vs.-rest classification** | **Prospective test dataset** | | | | |
| --- | --- | --- | --- | --- | --- |
|  | **Accuracy (95% CI)** | **Sensitivity (95% CI)** | **Specificity (95% CI)** | **AUROC (95% CI)** | **AUPRC (95% CI)** |
| **MOST vs. POST + BOST** | | | | | |
| MAE | 93.43 (89.40, 97.08) | 90.91 (83.82, 97.01) | 95.77 (90.78, 100.00) | 0.945 (0.898, 0.982) | 0.920 (0.841, 0.985) |
| DINO | 72.26 (64.96, 79.56) | 86.36 (77.48, 93.94) | 59.15 (47.86, 70.75) | 0.849 (0.778, 0.911) | 0.865 (0.792, 0.926) |
| EVA | 86.86 (81.02, 91.97) | 89.39 (81.54, 95.59) | 84.51 (75.36, 92.11) | 0.943 (0.897, 0.985) | 0.924 (0.866, 0.966) |
| iBOT | 75.18 (67.88, 82.48) | 78.79 (68.57, 88.24) | 71.83 (61.53, 81.95) | 0.844 (0.774, 0.906) | 0.869 (0.795, 0.923) |
| **POST vs. MOST + BOST** | | | | | |
| MAE | 94.16 (89.78, 97.81) | 80.00 (57.14, 100.00) | 95.90 (91.77, 99.17) | 0.887 (0.749, 0.990) | 0.730 (0.485, 0.921) |
| DINO | 88.32 (82.48, 93.43) | 46.67 (20.00, 72.73) | 93.44 (88.80, 97.56) | 0.823 (0.711, 0.922) | 0.520 (0.282, 0.744) |
| EVA | 90.51 (85.40, 94.89) | 73.33 (50.00, 93.75) | 92.62 (87.50, 96.72) | 0.892 (0.802, 0.987) | 0.734 (0.533, 0.900) |
| iBOT | 87.59 (81.75, 92.72) | 53.33 (27.27, 80.00) | 91.80 (86.51, 96.61) | 0.869 (0.785, 0.945) | 0.455 (0.259, 0.726) |
| **BOST vs. MOST + POST** | | | | | |
| MAE | 93.43 (89.05, 97.08) | 92.86 (85.45, 98.37) | 93.83 (88.46, 98.70) | 0.965 (0.931, 0.990) | 0.947 (0.885, 0.989) |
| DINO | 70.80 (62.77, 78.10) | 46.43 (32.77, 59.63) | 87.65 (79.76, 94.26) | 0.822 (0.748, 0.884) | 0.737 (0.616, 0.844) |
| EVA | 83.21 (76.64, 89.05) | 71.43 (58.46, 82.77) | 91.36 (85.54, 96.51) | 0.940 (0.901, 0.970) | 0.911 (0.844, 0.960) |
| iBOT | 74.45 (67.15, 81.75) | 60.71 (48.07, 72.58) | 83.95 (75.64, 91.36) | 0.806 (0.726, 0.876) | 0.672 (0.555, 0.812) |

Models pretrained with different self-supervised learning (SSL) approaches, including masked autoencoders (MAE), DINO, EVA, and iBOT, undergo the same fine-tuning processes for downstream tasks. The OSPM-enhanced classification model is pretrained with MAE. AUROC, area under the receiver operating characteristic. AUPRC, area under the precision-recall curve. CI, confidence interval. MOST, malignant ocular surface tumor. POST, premalignant ocular surface tumor. BOST, benign ocular surface tumor.

**Table S11. Performance of models using different deep learning architectures in** **the NEH internal test dataset.**

| **One-vs.-rest classification** | **NEH internal test dataset** | | | | |
| --- | --- | --- | --- | --- | --- |
|  | **Accuracy (95% CI)** | **Sensitivity (95% CI)** | **Specificity (95% CI)** | **AUROC (95% CI)** | **AUPRC (95% CI)** |
| **MOST vs. POST + BOST** | | | | | |
| OECM | 94.17 (89.32, 98.06) | 87.50 (73.08, 100.00) | 96.20 (91.36, 100.00) | 0.986 (0.967, 0.998) | 0.964 (0.908, 0.995) |
| ConvNeXt | 88.35 (82.52, 94.17) | 70.83 (52.38, 88.89) | 93.67 (87.82, 98.70) | 0.936 (0.885, 0.975) | 0.821 (0.675, 0.934) |
| DenseNet121 | 88.35 (81.55, 94.17) | 75.00 (56.00, 91.30) | 92.41 (86.06, 97.58) | 0.950 (0.905, 0.982) | 0.873 (0.742, 0.953) |
| **POST vs. MOST + BOST** | | | | | |
| OECM | 91.26 (85.44, 96.12) | 84.21 (66.67, 100.00) | 92.86 (87.01, 97.70) | 0.977 (0.951, 0.996) | 0.923 (0.823, 0.987) |
| ConvNeXt | 89.32 (82.52, 94.68) | 57.89 (33.33, 80.00) | 96.43 (91.86, 100.00) | 0.932 (0.876, 0.975) | 0.774 (0.596, 0.917) |
| DenseNet121 | 90.29 (84.47, 95.15) | 63.16 (40.91, 83.33) | 96.43 (91.95, 100.00) | 0.920 (0.828, 0.987) | 0.843 (0.686, 0.954) |
| **BOST vs. MOST + POST** | | | | | |
| OECM | 95.15 (90.29, 99.03) | 93.33 (86.73, 98.45) | 97.67 (92.50, 100.00) | 0.993 (0.980, 1.000) | 0.995 (0.985, 1.000) |
| ConvNeXt | 79.61 (70.87, 87.38) | 88.33 (79.34, 95.59) | 67.44 (52.27, 80.95) | 0.903 (0.842, 0.953) | 0.934 (0.884, 0.971) |
| DenseNet121 | 90.29 (84.47, 95.15) | 95.00 (88.14, 100.00) | 83.72 (72.09, 93.48) | 0.968 (0.934, 0.990) | 0.977 (0.952, 0.993) |

NEH, Ningbo Eye Hospital. AUROC, area under the receiver operating characteristic. AUPRC, area under the precision-recall curve. CI, confidence interval. MOST, malignant ocular surface tumor. POST, premalignant ocular surface tumor. BOST, benign ocular surface tumor. OECM, OSPM-enhanced classification model.

**Table S12. Performance of models using different deep learning architectures in the EHWMU external test dataset.**

| **One-vs.-rest classification** | **EHWMU external test dataset** | | | | |
| --- | --- | --- | --- | --- | --- |
|  | **Accuracy (95% CI)** | **Sensitivity (95% CI)** | **Specificity (95% CI)** | **AUROC (95% CI)** | **AUPRC (95% CI)** |
| **MOST vs. POST + BOST** | | | | | |
| OECM | 91.28 (87.92, 94.30) | 89.06 (80.37, 96.04) | 91.88 (88.36, 95.11) | 0.959 (0.931, 0.981) | 0.847 (0.744, 0.934) |
| ConvNeXt | 85.57 (81.54, 89.26) | 59.38 (47.62, 70.80) | 92.74 (89.13, 95.81) | 0.910 (0.874, 0.941) | 0.714 (0.597, 0.822) |
| DenseNet121 | 86.58 (82.89, 90.27) | 73.44 (62.60, 84.35) | 90.17 (86.21, 93.95) | 0.895 (0.847, 0.934) | 0.699 (0.580, 0.811) |
| **POST vs. MOST + BOST** | | | | | |
| OECM | 93.29 (90.27, 95.97) | 86.36 (75.76, 95.74) | 94.49 (91.46, 97.20) | 0.960 (0.919, 0.991) | 0.913 (0.843, 0.969) |
| ConvNeXt | 91.28 (87.58, 94.30) | 59.09 (44.71, 73.81) | 96.85 (94.50, 98.79) | 0.921 (0.868, 0.965) | 0.783 (0.667, 0.884) |
| DenseNet121 | 82.55 (78.19, 86.58) | 63.64 (48.78, 77.78) | 85.83 (81.67, 89.94) | 0.805 (0.706, 0.884) | 0.612 (0.447, 0.743) |
| **BOST vs. MOST + POST** | | | | | |
| OECM | 89.93 (86.24, 93.29) | 86.84 (81.94, 91.51) | 95.37 (90.94, 99.06) | 0.957 (0.928, 0.980) | 0.968 (0.941, 0.989) |
| ConvNeXt | 79.53 (74.83, 83.89) | 88.95 (84.21, 93.02) | 62.96 (53.15, 72.17) | 0.857 (0.812, 0.899) | 0.904 (0.859, 0.943) |
| DenseNet121 | 78.52 (73.49, 83.22) | 76.32 (70.29, 82.64) | 82.41 (74.78, 88.89) | 0.858 (0.812, 0.902) | 0.905 (0.862, 0.943) |

EHWMU, Eye Hospital of Wenzhou Medical University. AUROC, area under the receiver operating characteristic. AUPRC, area under the precision-recall curve. CI, confidence interval. MOST, malignant ocular surface tumor. POST, premalignant ocular surface tumor. BOST, benign ocular surface tumor. OECM, OSPM-enhanced classification model.

**Table S13. Performance of models using different deep learning architectures in the JEH external test dataset.**

| **One-vs.-rest classification** | **JEH external test dataset** | | | | |
| --- | --- | --- | --- | --- | --- |
|  | **Accuracy (95% CI)** | **Sensitivity (95% CI)** | **Specificity (95% CI)** | **AUROC (95% CI)** | **AUPRC (95% CI)** |
| **MOST vs. POST + BOST** | | | | | |
| OECM | 92.88 (90.03, 95.44) | 86.21 (78.69, 93.29) | 95.08 (92.47, 97.53) | 0.927 (0.892, 0.959) | 0.817 (0.729, 0.897) |
| ConvNeXt | 82.91 (78.63, 86.61) | 72.41 (62.50, 81.40) | 86.36 (82.13, 90.15) | 0.867 (0.820, 0.907) | 0.670 (0.570, 0.790) |
| DenseNet121 | 60.40 (55.27, 65.24) | 67.82 (58.12, 77.92) | 57.95 (51.93, 63.79) | 0.694 (0.630, 0.754) | 0.442 (0.349, 0.550) |
| **POST vs. MOST + BOST** | | | | | |
| OECM | 93.16 (90.31, 95.73) | 82.35 (68.00, 94.59) | 94.32 (91.57, 96.82) | 0.891 (0.797, 0.963) | 0.744 (0.595, 0.871) |
| ConvNeXt | 84.90 (81.48, 88.32) | 50.00 (32.37, 66.67) | 88.64 (85.26, 92.19) | 0.778 (0.680, 0.865) | 0.335 (0.208, 0.500) |
| DenseNet121 | 85.75 (82.05, 89.17) | 41.18 (25.58, 58.48) | 90.54 (87.26, 93.64) | 0.746 (0.638, 0.844) | 0.427 (0.264, 0.588) |
| **BOST vs. MOST + POST** | | | | | |
| OECM | 91.17 (88.03, 94.02) | 90.43 (86.62, 94.21) | 92.56 (87.44, 96.85) | 0.940 (0.910, 0.965) | 0.964 (0.941, 0.982) |
| ConvNeXt | 77.49 (72.93, 81.77) | 76.09 (70.45, 81.55) | 80.17 (72.31, 86.98) | 0.860 (0.817, 0.899) | 0.911 (0.866, 0.945) |
| DenseNet121 | 63.82 (58.40, 68.66) | 52.17 (45.70, 58.50) | 85.95 (79.13, 92.03) | 0.804 (0.753, 0.850) | 0.879 (0.835, 0.917) |

JEH, Jiangdong Eye Hospital. AUROC, area under the receiver operating characteristic. AUPRC, area under the precision-recall curve. CI, confidence interval. MOST, malignant ocular surface tumor. POST, premalignant ocular surface tumor. BOST, benign ocular surface tumor. OECM, OSPM-enhanced classification model.

**Table S14. Performance of models using different deep learning architectures in the prospective test dataset.**

| **One-vs.-rest classification** | **Prospective test dataset** | | | | |
| --- | --- | --- | --- | --- | --- |
|  | **Accuracy (95% CI)** | **Sensitivity (95% CI)** | **Specificity (95% CI)** | **AUROC (95% CI)** | **AUPRC (95% CI)** |
| **MOST vs. POST + BOST** | | | | | |
| OECM | 93.43 (89.40, 97.08) | 90.91 (83.82, 97.01) | 95.77 (90.78, 100.00) | 0.945 (0.898, 0.982) | 0.920 (0.841, 0.985) |
| ConvNeXt | 85.40 (79.56, 91.24) | 75.76 (65.51, 85.40) | 94.37 (88.41, 98.73) | 0.908 (0.847, 0.958) | 0.879 (0.786, 0.965) |
| DenseNet121 | 80.29 (73.72, 86.13) | 89.39 (81.03, 96.49) | 71.83 (62.22, 82.14) | 0.892 (0.833, 0.943) | 0.866 (0.776, 0.951) |
| **POST vs. MOST + BOST** | | | | | |
| OECM | 94.16 (89.78, 97.81) | 80.00 (57.14, 100.00) | 95.90 (91.77, 99.17) | 0.887 (0.749, 0.990) | 0.730 (0.485, 0.921) |
| ConvNeXt | 86.86 (81.02, 91.97) | 20.00 (0.00, 44.23) | 95.08 (90.83, 98.39) | 0.829 (0.695, 0.941) | 0.423 (0.240, 0.709) |
| DenseNet121 | 91.97 (86.86, 95.62) | 53.33 (26.95, 80.00) | 96.72 (93.19, 99.19) | 0.842 (0.688, 0.956) | 0.557 (0.308, 0.785) |
| **BOST vs. MOST + POST** | | | | | |
| OECM | 93.43 (89.05, 97.08) | 92.86 (85.45, 98.37) | 93.83 (88.46, 98.70) | 0.965 (0.931, 0.990) | 0.947 (0.885, 0.989) |
| ConvNeXt | 76.64 (69.34, 83.21) | 87.50 (77.59, 95.74) | 69.14 (59.26, 79.04) | 0.851 (0.783, 0.908) | 0.735 (0.615, 0.866) |
| DenseNet121 | 79.56 (72.99, 86.13) | 66.07 (53.45, 77.79) | 88.89 (81.50, 95.06) | 0.850 (0.774, 0.912) | 0.762 (0.636, 0.877) |

AUROC, area under the receiver operating characteristic. AUPRC, area under the precision-recall curve. CI, confidence interval. MOST, malignant ocular surface tumor. POST, premalignant ocular surface tumor. BOST, benign ocular surface tumor. OECM, OSPM-enhanced classification model.

**Table S15. Comparison of OECM with Ophthalmologists in the classification of malignant, premalignant, and benign ocular surface tumors.**

| **One-vs.-Rest Classification** | | **OECM** | **Senior 1** | **Senior2** | **Junior1** | **Junior2** | **Junior1 + OECM** | **Junior2 + OECM** | ***P1*** | ***P2*** | ***P3*** | ***P4*** | ***P5*** | ***P6*** |
| --- | --- | --- | --- | --- | --- | --- | --- | --- | --- | --- | --- | --- | --- | --- |
|  | **MOST vs. POST + BOST** | | | | | | | | | | |  |  |  |
| Accuracy (95% CI) | 90.7% (86.0-95.3) | | 91.3% (86.8-95.8) | 92.0% (87.7-96.3) | 79.3% (72.9-85.8) | 72.0%  (64.8-79.2) | 86.0%  (80.4-91.6) | 82.0%  (75.9-88.1) | 1.000 | 0.824 | 0.002 | 0.000 | 0.064 | 0.011 |
| Sensitivity (95% CI) | 88.0% (79.0-97.0) | | 88.0% (79.0-97.0) | 90.0% (81.7-98.3) | 64.0% (50.7-77.3) | 60.0%  (46.4-73.6) | 78.0%  (66.5-89.5) | 72.0%  (59.6-84.4) | 1.000 | 1.000 | 0.008 | 0.003 | 0.167 | 0.146 |
| Specificity (95% CI) | 92.0% (86.7-97.3) | | 93.0% (88.0-98.0) | 93.0% (88.0-98.0) | 87.0% (80.4-93.6) | 78.0%  (69.9-86.1) | 90.0%  (84.1-95.9) | 87.0%  (80.4-93.6) | 1.000 | 1.000 | 0.227 | 0.003 | 0.375 | 0.064 |
|  | **POST vs. MOST + BOST** | | | | | | | | | | |  |  |  |
| Accuracy (95% CI) | 90.0% (85.2-94.8) | | 89.3% (84.4-94.3) | 88.7% (83.6-93.7) | 68.7% (61.2-76.1) | 72.7%  (65.5-79.8) | 82.7%  (76.6-88.7) | 83.3%  (77.4-89.3) | 1.000 | 0.845 | 0.000 | 0.000 | 0.001 | 0.009 |
| Sensitivity (95% CI) | 82.0% (71.4-92.6) | | 80.0% (68.9-91.1) | 78.0% (66.5-89.5) | 52.0% (38.2-65.8) | 58.0%  (44.3-71.7) | 68.0%  (55.1-80.9) | 72.0%  (59.6-84.4) | 1.000 | 0.815 | 0.001 | 0.008 | 0.077 | 0.167 |
| Specificity (95% CI) | 94.0% (89.3-98.7) | | 94.0% (89.3-98.7) | 94.0% (89.3-98.7) | 77.0% (68.8-85.2) | 80.0%  (72.2-87.8) | 90.0%  (84.1-95.9) | 89.0%  (82.9-95.1) | 1.000 | 1.000 | 0.002 | 0.003 | 0.015 | 0.035 |
|  | **BOST vs. MOST + POST** | | | | | | | | | | |  |  |  |
| Accuracy (95% CI) | 94.0% (90.2-97.8) | | 95.3% (92.0-98.7) | 91.3% (86.8-95.8) | 76.0% (69.2-82.8) | 83.3%  (77.4-89.3) | 86.0%  (80.4-91.6) | 86.7%  (81.2-92.1) | 0.774 | 0.424 | 0.000 | 0.000 | 0.006 | 0.383 |
| Sensitivity (95% CI) | 92.0% (84.5-99.5) | | 96.0% (90.6-100) | 90.0% (81.7-98.3) | 70.0% (57.3-82.7) | 74.0%  (61.8-86.2) | 86.0%  (76.4-95.6) | 84.0%  (73.8-94.2) | 0.500 | 1.000 | 0.003 | 0.004 | 0.021 | 0.227 |
| Specificity (95% CI) | 95.0% (90.7-99.3) | | 95.0% (90.7-99.3) | 92.0% (86.7-97.3) | 79.0% (71.0-87.0) | 88.0%  (81.6-94.4) | 86.0%  (79.2-92.8) | 88.0%  (81.6-94.4) | 1.000 | 0.549 | 0.001 | 0.065 | 0.143 | 1.000 |

*P1* indicates the p-value calculated between OECM and senior1 using the McNemar test. *P2* indicates the p-value calculated between OECM and senior2 using the McNemar test. *P3* indicates the p-value calculated between OECM and junior1 using the McNemar test. *P4* indicates the p-value calculated between OECM and junior2 using the McNemar test. *P5* indicates the p-value calculated between the junior1 and “junior1 + OECM” using the McNemar test. *P6* indicates the p-value calculated between the junior2 and “junior2 + OECM” using the McNemar test. CI, confidence interval. OECM, OSPM-enhanced classification model. MOST, malignant ocular surface tumor. POST, premalignant ocular surface tumor. BOST, benign ocular surface tumor

**Table S16. The pretraining setting of OSPM.**

| **Parameter** | **Value** |
| --- | --- |
| Image size | 224 |
| Patch size | 16 |
| Mask ratio | 0.75 |
| Optimizer | AdamW |
| Base learning rate | 1.50E-04 |
| Weight decay | 0.05 |
| Optimizer momentum | β1,β2=0.9,0.95 |
| Batch size | 1536 |
| Learning rate schedule | Cosine decay |
| Warmup epochs | 40 |
| Total epochs | 800 |

OSPM, ocular surface pretrained model.
